# Smart Classrooms: How Sensors and AI Are Shaping Educational Paradigms

**DOI:** 10.3390/s24175487

**Published:** 2024-08-24

**Authors:** Xiaochen Zhang, Yiran Ding, Xiaoyu Huang, Wujing Li, Liumei Long, Shiyao Ding

**Affiliations:** Department of Industrial Design, Guangdong University of Technology, Guangzhou 510006, China; xzhang@gdut.edu.cn (X.Z.); 2112317010@mail2.gdut.edu.cn (Y.D.); 2112317043@mail2.gdut.edu.cn (X.H.); 3119008036@mail2.gdut.edu.cn (W.L.); 2112317082@mail2.gdut.edu.cn (L.L.)

**Keywords:** smart classrooms, educational technology, sensors, artificial intelligence, machine learning

## Abstract

The integration of advanced technologies is revolutionizing classrooms, significantly enhancing their intelligence, interactivity, and personalization. Central to this transformation are sensor technologies, which play pivotal roles. While numerous surveys summarize research progress in classrooms, few studies focus on the integration of sensor and AI technologies in developing smart classrooms. This systematic review classifies sensors used in smart classrooms and explores their current applications from both hardware and software perspectives. It delineates how different sensors enhance educational outcomes and the crucial role AI technologies play. The review highlights how sensor technology improves the physical classroom environment, monitors physiological and behavioral data, and is widely used to boost student engagements, manage attendance, and provide personalized learning experiences. Additionally, it shows that combining sensor software algorithms with AI technology not only enhances the data processing and analysis efficiency but also expands sensor capabilities, enriching their role in smart classrooms. The article also addresses challenges such as data privacy protection, cost, and algorithm optimization associated with emerging sensor technologies, proposing future research directions to advance educational sensor technologies.

## 1. Introduction

With the rapid development of information technology, the smart classroom, as an emerging educational model, is gradually becoming an important part of modern education. Smart classrooms are defined as technology-assisted, closed environments that enhance the teaching and learning experience [1]. The emergence of the “smart classroom” represents a paradigm shift in educational environments, merging traditional teaching and learning methods with advanced technological integration. As a model for contemporary educational settings, smart classrooms are often characterized by the use of digital tools, information and communication technologies (ICT), and interactive learning systems. Smart classrooms are designed to bridge the gap between students and teachers, to help teachers teach more effectively, and to make the environment more conducive to teaching and learning [2].

In recent years, smart sensor technologies have emerged as pivotal tools in education transformation. Sensor technology is an indispensable basic part of the application of many advanced technologies such as AI (Artificial Intelligence), intelligent learning technology, the Internet of Things, information technology, and big data in the classroom. In smart classrooms, the presence of sensors provides a way to naturally collect learning data during the learning process, forming the data foundation of intelligent systems, and providing educators with unprecedented opportunities to deepen students’ learning experience and improve the teaching efficiency.

The progress of Artificial Intelligence technology in recent years has been remarkable, and the tremendous impact of this technology is undoubtedly being introduced into smart classrooms. The introduction of AI combined with emerging technologies having the form of interactive, remote, and mobile computing in physical and/or virtual environments constitutes an evident trend in the development of the concept of the smart classroom [3]. There are also integrated applications of AI technology with sensor technology, where data collected by sensors can be processed and analyzed by AI algorithms. Spikol et al. points out that computer systems have been widely used with the delivery of instructional content, which are ideal systems for assisting in teaching and learning analytics [4]. Therefore, AI technologies such as big data analytics and machine learning methods can be deployed to help understand and categorize learning outcomes. Further integration and application of artificial intelligence and sensor technologies are foreseen.

The smart classroom is a research topic that extends multiple disciplines, and there is a considerable number of studies reviewing the progress of research in this area, whether from a technological dimension, pedagogical perspective, or sociological perspective.

Saini and Goel [2] described an interdisciplinary research on smart classroom technologies, dividing smart classrooms into “Smart Content”, “Smart Engagement”, “Smart Assessment”, and “Smart Physical Environment” to describe and review the technological research progress in smart classrooms. Saini and Goel also present potential challenges and future perspectives and recommendations, summarizing the technological development and applications of smart classrooms in a more comprehensive way. Alfoudari et al. [5], employing the systematic review approach, focus on the social and technological challenges faced by smart classrooms as well as future research directions at the macro level. For a more specific field of research, Wang et al. [6] presented a review of sensor technology in the classroom, focusing on devices and systems that use eye-tracking sensors to monitor student attention in smart classrooms. The advantages, characteristics and limitations of different eye-tracking devices and systems were illustrated in this review, providing a detailed introduction to the current technology of eye-tracking sensor systems. From the perspective of artificial intelligence, Zawacki-Richter et al. [7] summarized the application areas of AI technologies in academic support services, institutional services, and administrative services in higher education. In addition to a general overview of artificial intelligence in smart classrooms, Dimitriadou and Lanitis [3] conducted a comprehensive SWOT analysis of the advantages, disadvantages, opportunities, and threats of applying artificial intelligence in smart classrooms.

The research on the technology of smart classrooms and the application of AI technology in education (AIEd) has aroused greater interest in the research community; however, the research on the technology for sensors in smart classrooms is scattered and lacks systematic review and organization. Additionally, research for the integration of AI technology and sensor technology in smart classrooms is also insufficient. Few studies have been conducted to review and summarize the significant enhancements that AI technology brings to sensors.

Compared to the previous survey [2,5,6,7], this paper brings a multifaceted contribution by systematically narrating the research: (1) We present the technological applications and progress of smart classrooms from the perspective of sensor technology, comprehensively reviewing its applications and classifications in contexts such as monitoring students’ attention, taking attendance, and teaching assessment analysis in smart classrooms. (2) At the software technology level, we review the advancements in AI technology and introduce various applications of combining AI technology with sensors in smart classrooms. We explore how AI technology can support smart learning through its integration with sensors, and analyze and compare the roles and functions of different AI technologies in sensor technology. (3) We explore the potential challenges and risks associated with integrating sensor and AI technologies, and discuss possible future research directions. Our aim is to provide upcoming researchers with the necessary domain knowledge by illustrating the current state of the art and research trends in the application of sensors and their integration with AI technologies in smart classrooms, and to provide inspiration for future research.

The rest of this paper is organized as follows. In Section 2, we summarize the categories and trends of sensors in smart classrooms, and specify how environmental and biometric sensors can support smart classrooms, respectively. Section 3 analyzes and compares different AI technologies and describes how different AI technologies can enhance the performance and enrich the functionality of sensors. Section 4 discusses the current problems and challenges, as well as emerging directions for future research. Finally, the review paper is concluded with Section 5.

## 2. Smart Sensor Technologies and Applications

**Figure 1:**
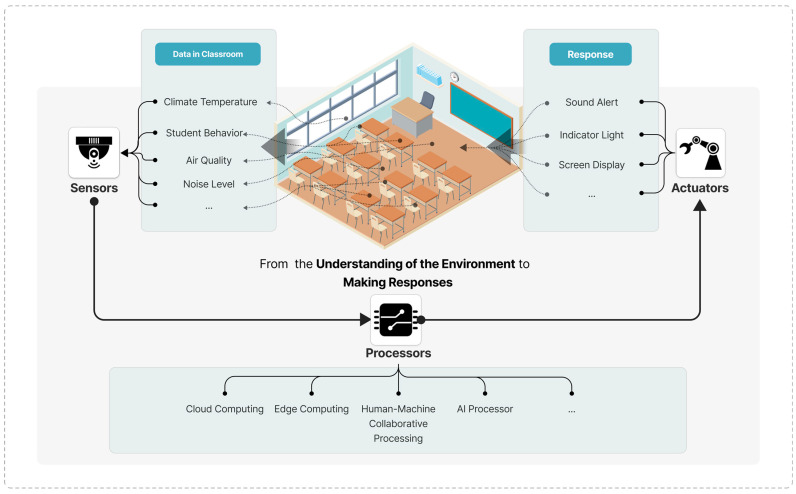
A representative example of a smart classroom sensor system consisting of (1) sensors that collect classroom environment and biological data, (2) processors that process data and make instructions (usually cloud computing, edge computing, human–computer collaborative processing, AI processors, etc.), and (3) actuators that receive instructions and respond to classroom feedback in the form of sound or indicator lights.

In the field of education, the application of sensor technology focuses on all aspects of learning and teaching. Shown in Figure 1 is a representative example of a smart classroom sensor system. This system includes sensors for multiple modality data collection, such as cameras, temperature and thermal sensors, light sensors, air quality, personnel recognition cameras, sound level meters, etc., which can capture human behavior and environmental characteristics as data reference opinions to create a good learning experience. This section delves into smart sensors’ diverse applications, highlighting the novel ways in which they contribute to education. This survey categorizes the sensors into environmental and biometric sensors, covering the breadth of sensor types and their direct applications in smart classrooms.

### 2.1. Environmental Sensors

Sensor technologies have been deployed to collect real-time measurements of physical environmental conditions. Table 1 summarizes typical studies of environmental sensors in smart classrooms. These systems are normally equipped with micro controllers to control actuators and data displays, making invisible phenomena visible and actionable. A Raspberry Pi-based Weather Management System (WMS) proposed by Hu and Huang [8] can be used to collect numerous weather data including temperature, humidity, and wind direction, and is a typical sensor system used in educational environments. Mendell and Heath [9] pointed out that the deployment of environmental sensors is able to help to regulate and control the indoor classroom environment in order to create a comfortable physical environment, which is necessary for efficient learning.

Saini and Goel [2] comprehensively categorized the elements of a typical classroom physical environment into the following areas: temperature, humidity, radiation, VOCs, NO_2_ (from burning fuel items), CO_2_ (mainly emitted by humans), airborne particles (such as coarse dust particles (PM10) and fine particles (PM2.5)), carbon monoxide (CO), sound level, audio noise level, and lighting. In this subsection, we divided the application of environmental sensors in smart classrooms into the following parts: climate control, lighting, and noise control.

#### 2.1.1. Climate Control

The climate factor refers primarily to the suitability of the air, specifically the temperature and humidity of the air and the level of pollutants in the air. The study by Chiou and Tseng [18] proposed a smart classroom management system deployed in a lab classroom environment. In this study, a Wireless Sensor Network (WSN) was created using Zigbee technology to enable the regulation of the physical environment of the classroom (temperature, humidity, lighting, etc.). A field experiment was conducted to verify the effectiveness of the proposed system. The experimental results showed that the system had good accuracy and robustness in a real-time environment. Stazi et al. [10] proposed a smart window opening and closing to improve air quality and thermal comfort in the classroom, using a PT100 thermistor sensor to measure the temperature and a CO_2_ sensor to monitor the air quality. The results of a comparative study in two adjacent classrooms showed that this system provided good quality in terms of indoor air quality, thermal comfort and user satisfaction. Twumasi et al. [13] utilized a passive infrared radio sensor to automatically start and turn off the fan in the classroom. When a student enters the classroom, the infrared energy emitted activates the PIR sensor and provides it to the microcontroller, which triggers a relay to “turn on” the fan, and ten minutes later “turn off” the fan when no motion is detected. The fan will only ‘turn on’ when the room temperature reaches 25 degrees Celsius to 30 degrees Celsius. In the research by Pastor et al. [14], the proposed system allows simultaneous real-time monitoring of multi-dimensional indicators including CO_2_, temperature, humidity, and particulate matter in shared public spaces in higher education settings. This system is able to automatically control the corresponding ventilation or air conditioner to regulate the air conditions.

To reduce the risk of infection to students, maintaining a healthy classroom environment during the COVID-19 pandemic is even more necessary. Infrared cameras are employed in [17] to monitor students for fever and can help regulate the temperature of the classroom. In addition, Deepaisarn et al. [19] proposed an end-to-end camera-based human physical distance recording system for indoor environments (especially classrooms). The recording system automatically tracks the location of students and the direction of their movement in the classroom. It also records the movement of students to and from their seats, helping to maintain physical distance between students indoors and reducing the risk of disease transmission.

#### 2.1.2. Lighting

In terms of classroom lighting, lighting in educational settings is no longer static now. Adaptive lighting systems, which adjust based on natural light and classroom activities, have been found to improve student alertness and reduce eye strain, thereby enhancing the learning experience. According to a study proposed by Du et al. [20], in terms of the psychological state of learning, the more comfortable the light environment is, the stronger the willingness to learn and the higher the enthusiasm for learning. After research, we found that current mainstream research focuses on several issues including reducing the waste of lighting resources and automatically adjusting indoor light intensity to enhance the lighting experience and effect.

The lighting on the surface of the desk calculations is noticeably uneven, which negatively affects the physical and mental state of the students. Amelkina and Duplenkova [15] developed a lighting control system with smooth control function, light sensors, and presence sensors, which provided a three-zone lighting intensity adjustment for each row of lighting equipment in the classroom. This arrangement would maintain the level and uniformity of lighting under combined lighting conditions and will greatly save energy. This article also found that, in the case of combined lighting, there is a significant non-uniformity in the calculated lighting on the desk surface, which can have a negative impact on students’ physical and mental states, which is consistent with the views of Du et al. [20].

Zola Cruz et al. [21] presented a prototype for automated lighting control and validated its effectiveness in a Mexican higher education setting. This system utilized a combination of PIR (Pyroelectric Infrared Sensors) and LDR (Light Dependent Resistors) within the IoT paradigm to automate lighting based on room occupancy and ambient light levels, thereby reducing energy consumption, management costs, and environmental impact. Furthermore, Chen et al. [16] proposed a classroom sub-area multi-mode lighting control system in higher education settings. In the design, an RS-485 communication network was employed to establish the lighting of each region. The program employed lighting sensors, vibration sensors, and infrared sensors to automatically gather information about the classroom lighting and work status and personnel distribution and to form a program controlled by this chip to realize the automatic switching of lights and achieve a good energy saving effect.

#### 2.1.3. Noise Control

Marques and Pitarma [11] pointed out that environmental noise had a direct impact on well-being and productivity. On one hand, high volume is associated with various health symptoms, such as high blood pressure and stress. On the other hand, sound comfort can improve concentration, communication, and productivity. This paper also introduced “iSoundIoT”, an IoT-based technology real-time noise monitoring system for indoor environments such as classrooms, comprising a calibrated sound sensor, a DFRobot gravity analog sound level meter, and a FireBeetle ESP8266 microcontroller system (DFRobot, Shanghai, China) to measure the sound level and to provide visual and audio alerts when predefined thresholds are exceeded. The system was also tested in the lab for two months using real-time continuous data collection, demonstrating its performance in improving acoustic comfort and well-being. Similarly, de Valencia et al. [12] employs a network of sensors (Arduino boards and sound sensors, specifically key-037 sensors) to capture and process real-time accurate noise level measurements and provide a visual representation of classroom noise by notifying teachers and students via visual indicators (led) in the system when the noise level exceeds a specific threshold, helping to create a quiet learning environment.

### 2.2. Biometric Sensors

In educational settings, biosensors also play a huge role in promoting the intelligence of classrooms. Biosensors are commonly used to monitor students’ or teachers’ physiological indicators, including expression, movement, eye gaze, body temperature, EEG signals, and heart rate. These sensors capture physiological and behavioral information, providing insights into student engagement, emotional responses, and cognitive processes. The applications of these sensors range from monitoring attention and stress levels to developing personalized learning pathways based on individual physiological responses. Moreover, biosensors enable accessibility support for students with disabilities. This subsection will demonstrate the applications of biosensors in smart classrooms according to several parts: engagement analysis, attendance, and accessibility support.

#### 2.2.1. Engagement Analysis

A high engagement state improves task performance and learning outcomes [22]. Learner engagement is influenced by a range of factors related to the individual learner, the task, and the learning environment [23]. The effectiveness of digital training can be enhanced by measuring and optimizing learners’ engagement during instruction [22]. After a survey of the literature, research on the monitoring of student engagement based on images is the most common, and this type of research mainly relies on camera sensors, eye tracking, or other image capturing devices.

##### Image-Based Biometric Sensors

Images contain a wealth of information, and there are many ways to analyze student engagement based on images. Verner and Dickinson [24] pointed out that the main indicators of student inattention in class are fidgeting, doodling, yawning, and looking around. To detect these indicators, numerous image-based studies have proposed methods to evaluate student engagements by analyzing facial expressions, eye gaze, body movements, and other indicators as shown in Table 2; among these, cameras and eye movement sensor devices to detect inattention are the more promising methods.

Cameras are capable of collecting image or video data of students’ posture, facial features, movements, and eye movements. In recent years, cameras technology has undergone significant developments. Camera hardware is becoming smaller and cheaper, which makes it easier to access. Meanwhile, software algorithm technology for processing images and videos has also been continuously enriched, especially the development of AI technology and machine learning technology.

To assess student engagement in STEM classrooms at U.S. universities, Alkabbany et al. [25] designed a biometric sensor network (BSN) consisting of a webcam, wall-mounted camera, and a high-performance computer. This system was designed to capture students’ head posture, eyes, body movements, and facial emotions. The recorded image features are used to train an artificial intelligence-based model to assess the behavioral and emotional engagement of students in a classroom environment. Four 75 min lecture experiments were conducted to compare the proposed technique with the state-of-the-art framework, and the results demonstrated that the proposed system showed superior accuracy in estimating behavioral and emotional engagement. Zhu et al. [26] developed a smart learning table based on visual sensors that can be used to identify abnormal sitting postures of primary and secondary school students aged 9–18 years old. The system optimized the recognition rate of abnormal sitting postures including long learning time, head tilt, body tilt, and head drooping to more than 92%. Moreover, this system is able to provide instant feedback and reminders when it detects a decline in student engagement and is able to prevent health problems due to sitting.

Thermal infrared imaging has been proved to be a reliable tool for non-invasive and non-contact assessment of vital signs, psychophysiological responses, and emotional states. Kim [31] utilized thermal infrared imaging to assess students’ psychological state in a Korean university classroom. The temperatures of each student’s area of interest (AOI) were collected and averaged to reflect the engagement of the entire class. The higher the temperature, the better the student’s classroom immersion. In Kim’s design, a mobile app was designed for teachers to display student engagement in the form of a traffic light, with green representing students immersed in the class, yellow representing average engagement, and red representing poor engagement.

Similarly, Hu et al. [28] proposed a method for identifying learning engagement in a VR environment based on multimodal feature integration. They employed HTC Vive Pro Eye as an eye-tracking device and HTC Vive Facial Tracker for facial tracking. These devices were connected to a computer and could simultaneously capture pupil diameter, eye gaze, and facial expression data. This study also adopted a head-mounted device with a ThinkGear ASIC module chip to integrate electroencephalogram data to collect brain signals, and used the data to evaluate learners’ attention in terms of cognitive, emotional, and behavioral performance in a VR environment. The entire experiment was conducted in an English course classroom for geography students at the university level. The results showed that the F1 score for learning concentration recognition using complete data input (including data of all types and dimensions) ranges from 0.66 to 0.73, which is significantly higher than the model using a single dimension or a single type of data.

An eye-tracking sensor is a device that monitors eye gaze position movement and blinking activity. The application of eye-tracking technology in multimedia learning research is gaining increasing attention [32]. Multi-sensor eye-tracking systems and hardware platforms have indeed become a fast and primary means of capturing and tracking eye movements, and have changed traditional teaching methods. Wang et al. [6] divided existing eye-tracking devices into several categories including tower-mounted eye trackers, screen-based eye trackers, head-mounted/wearable eye trackers, and mobile eye trackers. They also point out that head-mounted and mobile eye-tracking systems are more suitable for real-world applications and daily learning activities. Compared with tower-mounted eye trackers that require embedded cameras and forehead/chin rests, or screen-based eye trackers that require the use of display screens and have limited eye-tracking range, wearable and mobile eye-tracking systems are lighter, less burdensome, and have no monitoring range restrictions.

The main components of a head-mounted/wearable eye tracker include a scene camera sensor, an eye camera sensor, and a storage device. In a dual-eye-tracking investigation [30], Shvarts and Abrahamson utilized a head-mounted eye tracker: Pupil-Labs eye-tracking goggles. This device allowed the use of two people to move freely and simultaneously track the eye movements of a given shared environment. It was deployed to monitor the visual tracks of university psychology teachers and students, explore how the student and the tutor jointly focused on a specific visual object, and thus evaluated the interaction between the teacher and the student during the teaching and learning process. This technology offers a valuable tool for understanding the nuances of visual attention and interaction in educational settings. The article found that teachers’ perceptual activities were closely coordinated with students’ operational activities. Teachers were able to accumulate the experience of students’ learning process by observing students’ behavior and identifying the best time to conduct speech intervention. Zaletelj [33] adopted 2D and 3D features obtained from the non-intrusive Microsoft Kinect One depth camera sensor to characterize university students’ facial and body attributes, and estimated students’ attention levels in the classroom by analyzing gaze points, body posture, and several other behavioral parameters. The system used a Bagged Trees classifier and achieved an accuracy of 85.0% to 86.9% based on different parameters. The study also found that certain behaviors of students (such as writing, yawning, supporting the head, and gaze direction) were highly correlated with their attention levels. Similarly, Prieto et al. [34] also developed a wearable sensor system, mainly including SMI eye-tracking glasses and a smartphone with an accelerometer, to record the teacher’s gaze data (including the location and content of the teacher’s gaze in the classroom and audio data) as well as the teacher’s movement in the primary and secondary class, involving students aged 11 to 12, whether standing still or walking. The data were processed using a machine learning model to analyze the teacher’s behavior and interaction to fully understand the classroom dynamics and generate a visual choreography that can show the teaching activities and social interactions in the classroom over time. This method is particularly useful for educational research and teacher professional development, providing a new way to analyze and improve teaching strategies and classroom management.

The cost of existing commercial eye-tracking glasses (e.g., Tobii Pro Glasses 2, Pupil Labs Core, and SMI Eye Tracking Glasses) remains unaffordable for wide implementation in educational environments. Kassner et al. [29] proposed an open source, low-cost wearable eye-tracking solution, Pupil, which can serve as a low-cost alternative to commercial eye trackers.

Due to the increasing prevalence of personal mobile devices, mobile eye-tracking technology has become a low-cost alternative solution. This technology utilizes the front or rear camera of basic personal smart devices such as smartphones and tablets, in conjunction with powerful software applications, to achieve face detection, eye detection, iris or pupil detection, and gaze angle calculation [35]. This technology overcomes the high cost and limited mobility of existing commercial eye trackers, thereby providing a technical foundation for potential applications in education and classrooms.

However, it should be emphasized that most current research using eye trackers is conducted in a set eye-tracking laboratory. Long-term eye tracking in a real educational environment is truly original and novel [36]. We call for more research in real educational environments in the future to help understand the extent to which laboratory research content can be transferred to real educational environments.

##### No-Image-Based Biometric Sensors

Image-based sensors provide rich visual information in classroom; however, evaluating student engagement based on non-image biometrics (such as heart rate, sound, blood oxygen, EEG signals, and skin temperature) can also achieve outstanding monitoring results. Non-image biometrics are able to provide objective data for quantitative analysis. The relevant representative studies are summarized in Table 3.

Gligoric et al. and Basu [37,38] employed a basic microphone to collect data on classroom speech, to analyze and classify audio features including spectral entropy, formant frequency, autocorrelation, and energy, and to convert audio signals into a representation of the current level of students’ learning interest. This is achieved by combining machine learning algorithms. Advanced sensor technology can also facilitate the monitoring of student engagement through a multitude of modalities. Spikol et al. [4] utilized the Kinect One camera to monitor undergraduate engineering students’ facial and hand features while using the camera’s built-in microphone to monitor the sound level and frequency of students’ voices. Chiou and Tseng [18] proposed a smart classroom management system deployed in an experimental classroom environment. A Wireless Sensor Network (WSN) based on Zigbee technology was created in the paper, which used camera and microphone sensors to monitor students’ inattentive behaviors and alert them with LED lights and bracelet vibrations.

**Table 3:** Non-image-based biometric sensors for engagement analysis.

Sensor Type	Monitored Feature	Monitoring Purpose	Typical Studies
EEG Device	Brain activity	Concentration and mental state analysis	[39,40]
Acoustic Sensor	Voice modulation	Stress and emotional state analysis	[37,38]
Galvanic Skin Response Sensor	Skin conductance	Stress and emotional arousal	[41,42]
Heart Rate Monitor	Heart rate	Stress levels and engagement	[42,43]
Blood Oxygen Sensor	Blood oxygen levels	Health monitoring in physical education	[43]
Respiratory Rate Sensor	Respiratory rate	Stress levels and engagement	[40]

Emotions can be defined as voluntary or involuntary responses to external factors. People express their emotions through actions, such as speech, voice, facial expressions, and body language. However, the emotions expressed in such actions are sometimes manipulated and fail to clearly convey real feelings [44]. Monitoring objective physiological indicators can objectively and truly present the current state and concentration of learners in class. Hsu et al. [43] developed a reading attention monitoring system for e-book reading via computers in higher education smart classrooms. In addition to using the webcam on the display to monitor facial status, heart rate and blood oxygen sensors are installed on the mouse to collect heart rate and blood oxygen indicators. Combined with the Artificial Bee Colony (ABC) Algorithm, this system helps teachers understand students’ reading concentration rate in the classroom learning environment. Chen et al. [39] proposed an attention-based diagnosis and review mechanism (ADRM) based on EEG detection to help record passages where students have low attention levels in interactive English learning classes in a vocational high school. With printed textbooks and digital pens, targeted review can be carried out on the parts with low attention levels in subsequent learning. The experimental results showed that the review performance of the experimental group was significantly better than that of the control group, confirming that ADRM improves review performance. In addition, field-dependent learners performed better in review than field-independent learners. Moreover, learners with low ability were better in review performance in the experimental group than in the control group.

The monitoring of objective physiological data in the classroom often takes the form of wearable sensor devices, which monitors multiple modals of data. Carroll et al. [40] introduced a method for assessing unmanned aircraft systems (UAS) training classroom engagement using non-invasive physiological and behavioral monitoring technology. This was achieved by employing the Equivital EQ02 system to collect electrocardiogram (ECG), electrodermal activity (EDA), respiratory rate and movement acceleration measurements. Additionally, the VT3 mini eye tracker was employed to quantify the participant’s gaze position. The study showed that physiological and behavioral data can successfully classify learner engagement with 85% accuracy (including eye-tracking features) or 81% accuracy (excluding eye-tracking features). In addition, the study found that the use of low-invasive physiological measurements can observe changes in learner engagement in real time and can support teachers to adjust training in different learning situations to optimize learner engagement. Lascio et al. [41] adopted the Empatica E4 wristband with an electrodermal (EDA) activity sensor to monitor teacher and student heart rate and electrical skin activity during university lectures over three weeks in spring 2017. Results demonstrated the feasibility of using EDA sensors to monitor students’ emotional engagement during lectures, and that this technology can provide feedback to students and instructors to improve learning experiences and teaching methods. Similarly, Romine et al. [42] proposed a wearable educational health device, the Edu-fit tracker, which combines measurements of students’ electrodermal activity, skin temperature and heart rate to accurately track and record cognitive load during learning tasks. The monitoring of these physiological indicators can effectively reflect the changes in students’ cognitive load when dealing with different tasks, and predict students’ focus through machine learning technology. This system helped students manage and develop their own study habits and enhance their learning ability.

#### 2.2.2. Attendance

In the classroom, the traditional attendance method is a roll call, which is time-consuming, especially in large classrooms. Moreover, this method also easily leads to situations where someone substitutes for another person’s attendance. In recent years, skipping classes has become a common phenomenon in college classrooms, and it has spread, affecting the education and teaching of college classrooms. To solve this problem, many studies on automated attendance recording have emerged.

Currently, the main attendance methods include roll call, radio frequency technology (RFID [45], NFC [46]), smartphone-based technology (such as Bluetooth [47], NFC, and WIFI [48]), and biometric recognition technology (fingerprint features [49], facial features [50,51], and voice features [52]).

When using radio frequency and smart card technology to record attendance, students only need to bring the card close to the identifier to record their attendance. However, it also presents several issues such as the potential for card damage or loss, and the problem of students lending their cards to others to proxy attendance. In addition, some studies have developed some recognition applications based on the signal recognition of smartphone devices [47,48]. After entering a specific range, you can use Bluetooth, WIFI, or GPS technology on your personal mobile device to punch in and record, or scan the QR-Code to record. But notably, the use of smart devices is not allowed in some schools.

Attendance recognition based on biometrics can reduce the reliance on personal devices. Attendance management can be achieved by setting up recognition sensors in the classroom and combining them with automatic recognition algorithms. The basic process of attendance technology based on biometric sensors is: first, the basic biometric features of students are “registered” and input and stored in the database as templates. These features can be face, iris, voice, and fingerprint. Next is the verification stage, where the biometric features collected in real time are compared and matched with the templates in the database. If the pairing is successful, the recognition record is recorded as a successful attendance record.

After reviewing, we found that there exists diverse studies and methods that can help achieve attendance in the classroom, as shown in Table 4. Specifically, Ni et al. [50] proposed a higher education classroom roll-call system based on face detection technology using a camera as a sensor and the latest deep learning algorithm Faster-R-CNN, which can help quickly count students’ attendance status. In this system, a camera placed in front of the classroom collects classroom images and sends them to the school server for facial data analysis. After processing, the student’s attendance record can be obtained. Experimental results showed that the attendance rate of university classes had increased by 15.3% after using the roll call system based on facial detection technology. It also greatly saves class time, and the time required for roll call has been reduced by more than 10 times. Similarly, a webcam with a Haar-Cascade facial recognition classifier deployed on a chat robot was used to record attendance in [51]. After capturing the student’s facial image, it was identified and compared with the known student facial database registered in the system. The recognition result can also be prompted through the robot’s built-in speaker.

Fingerprints are also an important biological method for attendance. Gagandeep et al. [49] utilized an R305 optical fingerprint scanner to quickly process image recognition fingerprint, Wi-Fi module (ESP8266) to connect the device to the client application and ARM Cortex M3 microcontroller as the device control center; each student was assigned a unique ID number when building the database, which was compared with the optical fingerprint scanner scan to complete the personal identity verification. The work in [55] was also based on fingerprint recognition technology, and used a fingerprint student attendance information system model that met the examination needs. The above two studies merely proposed the design of the system but lacked sufficient experimental verification. With the development of smartphone technology, Adal et al. [56] recommended using smart mobile phones for fingerprint recognition attendance because most of these communication devices are now equipped with built-in fingerprint sensors which are much cheaper.

Voice recognition can also be used to record attendance. Amri et al. [52] introduced an attendance system based on voice biometrics. First, students were required to register and save their voices. Then, the power spectral density (PSD) and transition parameter methods were used to extract features from these voice samples to form a voice feature database. The real-time voice input by students was compared with the pre-registered voice data to identify the students, achieving an accuracy rate of 60%, which is potentially effective. The system introduced in [57] which utilized the built-in microphone of an Android smartphone for voice recognition can also be used for classroom management and attendance. However, the above two studies proposed pioneering solutions but lacked more rigorous empirical evidence. In addition, it is worth noting that almost all voice-based attendance systems use the built-in microphone of the mobile phone to record voice [58] because smartphones have built-in sound sensors, which are easy to use and do not require additional deployment.

Additionally, there are more specific methodologies that can be employed. He et al. [53] designed a smart chair system that can detect whether someone is sitting on the chair by installing an Interlink 402 pressure resistor on the chair and binds the chair to the student ID to check student attendance. The pressure data will be transmitted to the cloud to achieve real-time monitoring of the chair occupancy status.

In order to improve the attendance effect, attendance can also be multi-identified. Sarker et al. [59] proposed a multi-step authentication intelligent attendance management system that integrates radio frequency identification, a biometric fingerprint sensor, and password-based technology to reduce the number of substitute attendances. Yadav et al. [54] pointed out that the traditional attendance method was to call the student’s name or use a sensor-based card (RFID sensor) or biometric fingerprint-based attendance system. These methods are not efficient enough and cannot determine whether the student has attended the entire course. This study proposed a dynamic attendance management system that adds an infrared-based motion sensor to the basic recognition system. When the student’s movement is detected, the camera is activated to start recording video to identify the student, keep track of the student’s entry into and exit from the classroom, and determine whether the student has fully participated in the course. In order to solve the problem of absenteeism, Veer and Momin [60] also suggested tracking video frames to achieve a continuous or regular observation of student facial images, ensure the student’s attendance time, and reduce the number of students absent from the course. However, further validation of the system mentioned above is missing in their study.

#### 2.2.3. Accessibility Support

The application of sensor technology has enabled people with disabilities to gain additional abilities to understand the world, compensated for the body’s defects in “perception”, and enabled students with disabilities to engage in more diverse learning. This is the significant impact of the development of sensors.

For individuals with hearing and speech impairments, sign language represents the sole means of communication with non-disabled individuals. However, it is not necessary for non-disabled individuals to master sign language, as they lack the requisite knowledge. The application of image-based gesture recognition technology in smart classrooms has the potential to address this issue. Traditional gesture recognition devices and algorithms are often static and therefore cannot be applied to dynamic interactions in practice. Varshin and Vidhyapathi [61] proposed a dynamic finger gesture recognition device and algorithm based on the Microsoft Kinect device. The results of real-world tests showed that the system can recognize dynamic gestures of one hand and two hands, process depth data in real time, continuously monitor finger movements and quickly output results, facilitating the deployment of gesture recognition technology in real-time field environments, such as classrooms. Based on this technology, this system can provide powerful communication support for people with hearing and speech impairments in the classroom.

Zhang et al. [62] proposed a more efficient and simple smartphone-based gesture recognition system (GazeSpeak) that can interpret eye gestures in real time. The article also conducted a comparative experiment with the e-tran board. The results showed that the system was superior to the e-tran board in terms of communication speed and availability, showing good user satisfaction.

Lathière and Archambault [63] employed basic microphone sensors combined with speech recognition systems to convert sound into text; deaf and hard of hearing students were able to use subtitles to learn in class and understand the professor’s speech.

In early 2005, a software program called EyeDraw was developed for children with severe motor disabilities in [64], which runs on a computer with an eye-tracking device. This technology enabled disabled children to draw pictures by moving their eyes. The validation experiment found that, compared with EyeDraw Verson 1, the functionally improved EyeDraw Verson 2 can support all stages observed in the natural drawing learning process better. In addition, the study also found that adding features such as color, pattern, and sound feedback can improve the user experience and help attract users to use the software more deeply.

### 2.3. Overview of Sensor Technologies and Applications in Classroom

The role of smart sensors in the classroom is multifaceted. They can be employed to monitor the classroom environment and create a more conducive teaching environment. Additionally, they can serve as an assistant to educators, enabling them to sense students’ participation in real time and assist with recording students’ attendance, thereby alleviating the burden of classroom management for educators. This section introduces the types of sensors in smart classrooms and their various applications.

This study also revealed potential trends. (1) An increasing number of new sensor devices are being introduced into smart classrooms. Some of these are commercial biosensor products, which have the characteristics of miniaturization and wearability. For example, head-mounted eye trackers, brain wave sensors, skin electrical sensors, and other human factors testing wearable kits can provide objective physiological signal data to support quantitative analysis. Furthermore, some research devices are capable of monitoring multiple physiological signals simultaneously, as exemplified by the Equivital EQ02 [27]. (2) The increasing prevalence of smart mobile devices has led to the introduction of smaller and more integrated sensors into the classroom. In instances where students are permitted to utilize smartphones, these devices have also emerged as a cost-effective, portable and efficient form of sensor in the classroom. Teachers and students can employ smartphone cameras, microphones, fingerprint recognition sensors, accelerometers, and other sensors to monitor attendance and participation. Of course, the dominance of traditional sensors, such as cameras, microphones, and environmental sensors, in smart classroom applications has not been entirely supplanted by the advent of new technologies. Image cameras continue to provide the most abundant and intuitive information. (3) Moreover, the utilization of sensor technology in educational settings is indicative of a growing trend towards data fusion and multimodal analysis. There is an increasing emphasis on the integration of multi-sensor data, as opposed to relying on a single sensor. The formation of a comprehensive analysis enables a more accurate understanding and prediction of students’ learning status and needs.

## 3. Software: Integration with Artificial Intelligence

**Figure 2:**
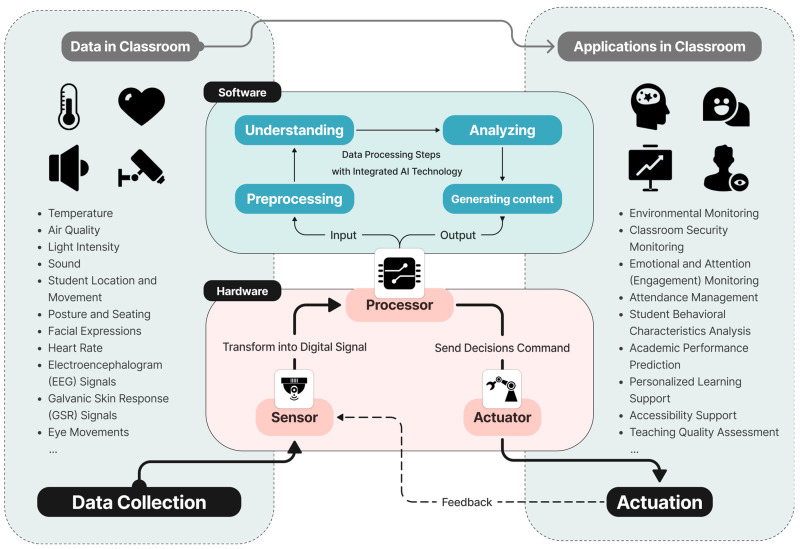
An AI-integrated sensor closed-loop system in a smart classroom. The classroom data collected by the sensors is sent to the processor integrated with AI technology, and instructions are generated through four steps including preprocessing, understanding, analyzing, and generating content to achieve more efficient and intelligent functional applications in the classroom.

The advancement of sensor technology in smart classrooms is contingent upon the development of hardware and the implementation of software algorithms. In particular, the integration of artificial intelligence technology enables sensor systems to process and analyze data with greater efficiency, thereby enabling the generation of more nuanced and personalized content, which in turn facilitates the delivery of higher quality educational services. In systems comprising sensors, actuators, and processors (as shown in Figure 2), the primary function of software algorithms is to (1) filter the data collected by the sensors, (2) comprehend the data, (3) analyze the data, (4) generate content, and (5) output content to the actuators.

Over the past decade, advancements in traditional algorithms and the emergence of novel AI technologies have led to notable enhancements in sensor software technology. Various branches of AI, including machine learning, natural language processing, and reasoning and judgment systems, have been investigated and implemented in the domain of education. This section will delineate the advancements in sensor technology within the context of education, with a particular focus on the role of software algorithms, particularly AI technology.

Today, artificial intelligence (AI) technology is defined as the technology and science that enables computer systems to perform tasks that normally require human intelligence. These tasks include, but are not limited to, learning (acquiring information and applying rules to use the information), reasoning (using rules to reach approximate or definite conclusions), self-correction, and understanding language.

Baker and Smith [65] provided a comprehensive definition of artificial intelligence, which they define as a computer that performs cognitive tasks, usually associated with human thinking, especially learning and problem solving. They also highlight that artificial intelligence is not a single technology but rather a general term for a range of technologies and methods, including machine learning, natural language processing, data mining, neural networks, and algorithms.

The capacity of AI to analyze vast quantities of data and automate complex tasks has opened up new avenues for enhancing both teaching and learning experiences. In the field of education, the application of AI, or Artificial Intelligence in Education (AIEd), has also become one of the emerging and rapidly developing fields. The survey [7] put forth four principal domains of AI implementation in higher education: (1) analysis and prediction, (2) evaluation and assessment, (3) adaptive systems and personalization, and (4) intelligent tutoring systems. The article by Silva et al. [66] posited that artificial intelligence (AI) in education serves as a tool to support various aspects of teaching practice evaluation, student learning performance prediction, student behavior analysis, and learning emotion recognition. AI tools for teachers are employed to automate the management of classroom attendance and student engagement, facilitate the evaluation and provision of feedback on student learning outcomes, and reduce the workload of teachers. System-oriented AI tools assist institutional managers in the management and monitoring of their institutions, providing information such as school staff flow.

From the perspective of combining with sensor technology, this article identifies four principal areas in the application of AI sensor technologies in education: analysis and prediction, teaching evaluation and learning feedback, personalized learning support, and teaching management. This section emphasizes the synergy between sensors and advanced AI computational technologies, illustrating how they work together to enhance educational experiences through data processing and understanding algorithms and data analysis and content generation algorithms.

### 3.1. Data Processing and Understanding

Processing and understanding the data collected by sensors is one of the main tasks of software algorithms. This enables the processor system to convert the physical information of sensors into data signals with recognizable value, realize the understanding and recognition of scenes, and provide a prerequisite for microprocessors to make decisions. After a literature survey, the research on data recognition algorithms based on AI technology is mainly applied to the understanding of biological characteristics such as teacher or student behavior and characteristic status. Compared with the understanding of the physical environment characteristics of the classroom, it is more complex and requires processing larger and more complex data.

The progress of AI technology in this field is particularly reflected in the large-scale application of machine learning technology, especially the deep learning technology that has developed rapidly in recent years. Deep learning technology has great potential in automatic feature processing, large-scale data recognition, generalization ability, data processing rate and efficiency, and accuracy in specific scenarios. It is able to realize end-to-end learning and direct training from input to output. Moreover, it has good performance in complex image recognition and audio processing.

At present, there are numerous significant studies on smart classroom image recognition algorithms. Meanwhile, traditional machine learning algorithms can also meet the requirements for data processing and achieve favorable outcomes when the amount of data and computing resources are limited. As an example, when dealing with linear problems such as EEG signals, body temperature, and ECGs on students, traditional machine learning algorithms are the simpler, easier to debug approach compared with deep learning algorithms.

Considering the prominent number and progress of classroom image recognition studies based on AI technology, this section will consider whether or not image recognition is a benchmark for illustrating the role of AI technology in the advancement of recognition algorithms from two perspectives: image-based and non-image-based recognition algorithms.

#### 3.1.1. Image-Based Recognition Algorithm

Images can present a wealth of information. The recognition and understanding of images can effectively help assess students’ emotional state, participation, attendance, and learning behavior patterns, and realize automated classroom management. Although existing devices such as electroencephalograms (EEGs), electrocardiograms (ECGs), and eye-tracking devices can be used to recognize emotions, cameras are the most promising type of sensor because visual images often have the richest information and do not need to be worn [67]. Typical image-based recognition algorithms, including the recognition of facial and body images are shown in Table 5.

Tarnowski pointed out that there are many ways to identify personal emotions (such as monitoring eye movements, body posture, electromyographic signals, heartbeat, etc.) [76], among which emotion recognition based on facial image features is the most commonly used method. Facial image recognition is also an effective method for automated attendance in classrooms. Lek and Teo [77] divided the FER (Facial Emotion Recognition) algorithms into traditional FER and FER based on deep learning. The methods used in traditional facial recognition technology include the Viola-Jones algorithm, Support Vector Regression (SVR), Support Vector Machine (SVM), Decision Tree, Random Forest (RF), Naive Bayes, K-Nearest Neighbors (KNN), Adaptive Boosting (AdaBoost), and other traditional machine learning technologies. Facial recognition algorithms based on deep learning technology mostly use the emerging machine learning technology of the Deep Neural Network (DNN). The main difference between them and traditional recognition algorithms is whether deep learning technology is used.

**Figure 3:**
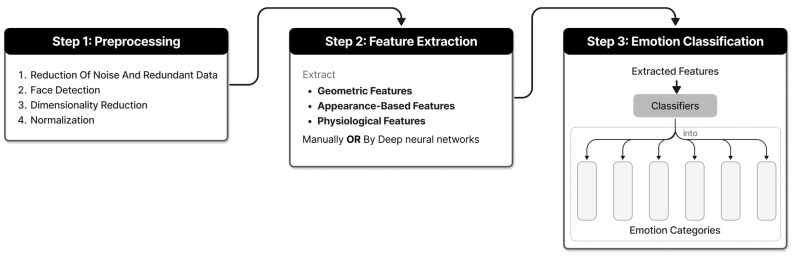
Process of facial emotion recognition.

Facial emotion recognition can be divided into the following steps, as shown in Figure 3: (1) Preprocessing: This involves the reduction of noise and redundant data, face detection, dimensionality reduction, and normalization. (2) Feature extraction: This includes the extraction of geometric features, appearance-based features, or physiological features of FER. (3) Emotion classification: This is achieved through the use of different classifiers to classify the extracted feature expressions into appropriate categories, thus enabling the identification of emotions. Within traditional FER algorithms, facial emotion expression features are extracted manually and then classified using non-deep neural network machine learning algorithms. In contrast, deep learning-based FER automatically extracts features and classifies them automatically, without the need for manual feature extraction. Deep neural networks are responsible for feature extraction and classification. Both methods can achieve good recognition efficiency in specific scenarios. Currently, support vector machines (SVMs) are a conventional learning emotion classifier that is widely used in FER systems. Besides, convolutional neural networks (CNNs) are the most commonly used deep learning classifiers. Lek and Teo [77] pointed out that the majority of literature studies employ CNNs in the feature extraction stage. This conclusion can also be preliminarily observed in Table 5.

Sabri et al. [68] employed the SVR (SVM regression) classifier of traditional machine learning technology to monitor the four emotions (happy, normal, sad, and surprised) of students engaged in online learning during the epidemic. The accuracy rate achieved was 99.16%. The application analyzed static frontal facial images of students to identify the emotion type. The specific process is as follows: the grayscale conversion and contrast stretching of the collected images are preprocessed, then the Haar Cascade or Viola-Jones algorithm is used for face monitoring to determine whether there is a face in the image. The face model technique is then employed for eye and mouth localization, the skin-color segmentation technique is used for image segmentation, and the Grey-Level Co-Occurrence Matrix (GLCM) is used for feature extraction. Following the aforementioned steps of image processing and feature extraction, the SVM regression classifier is employed for emotion classification. A smart classroom learning status management system is proposed in study [78]. It utilizes a range of sensor technologies, including cameras, body temperature sensors, pulse sensors, and image recognition technologies, to detect and collect a multitude of data points about students. This information is then processed through a Bayesian classification network, which is used to infer the students’ learning status. Furthermore, the system incorporates a feedback mechanism that not only furnishes the outcomes of immediate learning status analysis to educators but also alerts students who are identified as inattentive in class.

As for face recognition based on deep learning technology, the convolutional neural network (CNN) is the most widely used classification algorithm today. It can be implemented directly on the input image without using any face detection or feature extraction algorithm, which makes it the most effective algorithm [79]. The FER method based on deep learning significantly reduces the reliance on facial physics-based models and other pre-processing techniques by implementing “end-to-end” learning directly from the input image in the pipeline. As a distinct type of deep learning, CNN visualizes the input image to facilitate the comprehension of the model learned through various FER datasets and to demonstrate the emotion detection capability of the network trained on the dataset and various FER-related tasks [79]. Lasri and Solh [80] achieved 70% accuracy using Haar Cascades face detection with normalization and emotion recognition using CNNs on the FER 2013 database, with the data classified into seven facial expressions: surprise, fear, disgust, sadness, happiness, anger, and neutrality. The results demonstrate that facial emotion recognition is a feasible educational tool that can assist teachers in modifying their expressions according to students’ emotions.

Some studies have improved and adapted CNN to more diverse application scenarios. CNN can effectively help emotion recognition in static face images, while real-time face recognition needs to solve the delay problem. Due to the generation of millions of parameters, the delay of the hardware constraints used in the project is very large. In [69], a real-time classroom evaluation system was designed using computer vision target recognition technology. By removing the fully connected layer and combining the depth separable convolution with the remaining modules, a real-time emotion recognition model was established. Compared with the original model, the modified model reduced the parameters by 80 times, increased the recognition time by 1.5 times, and increased the average recognition accuracy (mAP) from 65.4% to 70.1%. It can realize real-time dynamic evaluation of students’ classroom performance and provide quick feedback to teachers.

CNN is commonly used for image recognition that requires large label training, but for scenarios with limited training data, the application of CNN has some limitations. In [70], the pre-trained CNN was fine-tuned and a two-step method combining data enhancement and CNN transfer learning was used to develop an automated attendance system focused on single-sample face recognition. After comparing five pre-trained models, DenseNet121 was found to be the best model for practical problems (up to 99% top-1 accuracy).

The Multi-Task Convolutional Neural Network (MTCNN) is a modified CNN, a deep cascaded multitask network that uses the intrinsic correlation between face recognition and matching to improve performance [81]. Specifically, three cascaded networks are used. These three cascaded networks are the Proposal Network (P-Net) for fast candidate window generation, the Refinement Network (R-Net) for high-precision candidate window filter selection, and the Output Network (O-Net) for generating the final wraparound box with key points of the face [82]. The MTCNN algorithm is widely used in face detection because of its high accuracy and fast detection speed. Wang et al. [71] employed an improved MTCNN algorithm for face detection and then the FaceNet model for recognition. The system achieved 98% accuracy in face recognition and 92% accuracy in student emotion recognition. The proposed method can realize students entering classroom check-in within 2s, which effectively improves the efficiency of classroom check-in, monitors the teaching process, and manages the teaching effect.

Current deep learning methods mainly focus on global or local facial features, while ignoring the multi-regional synergy of facial expressions from coarse to fine and the subtle variance of expressions [83]. To solve this problem, Guo et al. [84] proposed a multi-region attention transformation framework (MATF), which mainly includes a face local segmentation network, an attention transformation network, and a feature weight allocation mechanism. It associated global facial features with local details through the multi-dimensional joint method of FER, and integrated global and local facial details for expression recognition.

You Only Look Once version 5 (YOLOv5) is a CNN-based technology which is also widely used in image recognition technology. Hu et al. [72] introduced a power IoU loss function to You Only Look Once version 5 (YOLOv5) to detect students in You Only Look Once version 5 (YOLOv5) based on non-intrusive classroom videos and obtained 95.4% accuracy, and also developed a bimodal learning engagement detection method based on ResNet50 and CoAtNet, which combined with the use of KNN classifier obtained an accuracy of 93.94%.

Existing facial recognition algorithms are based on a single frontal image and are less effective in processing multi-faceted images in real classroom environments (e.g., low video resolution, blurred images, and less feature information). Therefore, detecting small faces becomes a challenging problem. Bie et al. [73] improved YOLOv5 with the concept of feature enhancement (FE-YOLOv5). In this study, Resnet-34_Focal was employed as the expression classification network, and the proposed upsampling module and Convolution-Batch Normalization-Leaky ReLU (CBL) module integrated more feature map information. The UPS module reduced the local perception field of the network, enabling the backbone network to learn detailed information more effectively. The CBL module accelerated the convergence of the model and improved the nonlinearity of the features, thereby achieving efficient feature extraction and fusion. This is more suitable for small face detection in classroom situations and solves the problem of the inaccurate recognition of small targets in the original network. In comparison to the original YOLOv5 algorithm, the average accuracy mAP of this method has increased by 7.18%, reaching 81.42%.

In the work by Zhang and Cao [85], different convolutional neural networks were employed. The system comprised MTCNN for face detection and an improved CNN for face recognition, as well as a memory-augmented neural network (MAN) for tracking students’ knowledge and learning status. These components were integrated to construct a multi-functional intelligent education system based on deep learning algorithms, which was capable of performing the four key functions: class attendance tracking, class status monitoring, knowledge status monitoring, and learning report analysis. The accuracy of face recognition was 96–97%, and the execution time of the model was less than or equal to 3 s.

In addition to the traditional FER algorithm and the deep learning FER algorithm, studies have been conducted that combine the two methods to perform student facial recognition. This algorithm is referred to as a hybrid facial recognition algorithm (Hybrid-FER). Rao [86] introduced a hybrid convolutional neural network (CNN) model that employs both manually designed features and features extracted from CNNs to identify the cognitive state of online e-learners during the COVID-19 pandemic. The model achieved an accuracy rate of 99.95% when the CK+ dataset was combined for training and testing. Shi et al. [74] developed a model for detecting confusion emotions generated by students in online learning. In the recognition method section, multiple methods (Histogram of Oriented Gradients (HOG), Local Binary Patterns (LBP), Support Vector Machine (SVM), and Convolutional Neural Network (CNN)) were combined to form four methods: HOG–SVM, LBP–SVM, CNN, and CNN–SVM. The CNN–SVM combination demonstrated the most promising performance, with average accuracy of 93.8%.

Facial recognition is the most prevalent method for assessing student status based on images in smart classrooms. Additionally, studies have indicated that the recognition of student body posture, head movement, hand movement, gaze, and other features can also present a wealth of visual information. In the study by Spikol et al. [4], traditional machine learning and deep learning algorithms were employed to perform multimodal sensor data recognition. The distance between the hand and the face, hand movement, and speed were utilized as a crucial feature to facilitate the monitoring and prediction of the success of the student team in open-ended tasks. In addition to the use of depth cameras, the study employed marker wristbands to obtain three-dimensional positional data regarding the hands of the students. Based on the aforementioned three-dimensional information, two key indicators can be calculated: the distance between the students’ hands and the distance between the hands and the face (which helps to understand the students’ current interaction, communication, and cooperation) and the hand movement speed (which, to a certain extent, reflects the students’ current activity and participation). The paper compares the performance of traditional machine learning and deep learning in multimodal analysis. Both algorithms can achieve data classification. The deep learning model has greater potential when using larger time windows and multimodal features.

Qi et al. [75] constructed a cascade analysis network model that integrates gaze estimation, facial expression recognition, and action recognition to identify students’ attention and engagement, thereby evaluating the engagement of students in online classes. In addition to recognizing students’ facial expressions, the convolutional neural network (CNN)-based L2CS-Net model is also used to identify students’ gaze directions, such as yaw and pitch angles, to help determine whether students are focusing on the screen. When students’ gaze deviated from the screen, the camera above the computer screen collected students’ movements and used an improved 3D convolutional neural network (Inflated 3D ConvNet) to process the image. This method stacked multiple consecutive video frames and used a cube-like convolution kernel to capture action features in temporal information. It also divided students’ actions into active actions, such as writing, reading, and passive actions, including eating, looking around, sleeping, and playing with mobile phones. The improved CNN-based algorithm proposed in this paper achieved an accuracy of 89.5% in the recognition task, which was higher than the traditional algorithms (such as LRCN, C3D, and Two Stream). Furthermore, it achieved a high-precision average angle error of 3.96° and 3.92° in student gaze estimation.

Monkaresi et al. [87] estimated students’ heart rates by analyzing small color changes in facial videos (based on photoplethysmography, PPG, a light volume tracing technique) and analyzed learners’ facial and heart rate features using traditional machine learning classifiers such as Bayesian networks, random forests, and logistic regression, which were used to assess students’ engagement. The results indicate that the accuracy of facial expression is superior to that of heart rate.

#### 3.1.2. Non-Image-Based Recognition Algorithms

Sensor algorithms for the recognition and understanding of image data are capable of parsing rich visual image information. However, image information alone may be somewhat one-dimensional, and non-image information can also provide reliable and efficient information that enriches the pathway to understanding student states. Table 6 summarizes algorithms for non-image-based recognition. AI technologies have also made great strides in helping to process and understand this kind of data, helping to broaden the ways and dimensions of understanding student states in smart ways and dimensions of understanding student states in the classroom.

**Figure 4:**
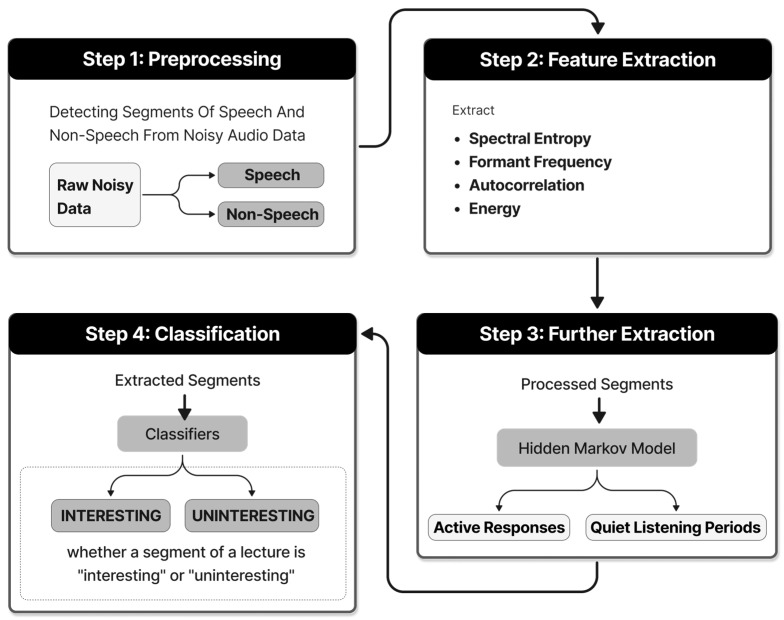
Process of speech emotion recognition.

Speech emotion recognition is a technique that can be used to analyze and predict the current interest level in the classroom, helping to improve the interaction between educators and learners. Existing studies have used diverse machine learning methods to achieve an understanding and recognition of audio. The study by Gligoric et al. [37] based on the Adaboost M1 machine learning algorithm transforms the sound signals collected by the sensors into an understanding of the interest level. The specific steps are shown in Figure 4. The sound is pre-processed using the algorithm proposed by Basu et al. [38] to detect talking and non-talking segments in noisy environments, and then key features are extracted from the processed sound segments, which mainly include the following:

- Spectral Entropy: a measure of the randomness and complexity of the sound signals, which is usually used to distinguish between voiced (such as speech) and unvoiced (such as breathing or background noise) sound.
- Formant Frequency: relates to the frequency characteristics of a sound and can reflect the quality of a vowel, helping to identify the content and intensity of speech.
- Autocorrelation: used to analyze the periodicity of a sound signal and helps to identify the rhythm, rate, and repetition pattern of a sound.
- Energy: the loudness of the sound signal, which reflects the level of activity and engagement of the students.

The sound signals were then further analyzed and classified again using the HMM (Hidden Markov Model) to distinguish between active student responses (such as questioning and discussion) and quiet listening periods, extracting more information about interest levels. Finally, these extracted features are fed into the Adaboost M1 weak classifiers for iterative recognition and comprehension, and the weighted vote of each weak classifier determines the final classification decision, which determines whether a segment of the lecture is “interesting” or “uninteresting”. The final classification decision is whether a segment of a lecture is “interesting” or “uninteresting”. It is worth mentioning that the use of the Adaboost M1 machine learning algorithm in the paper also helps to analyze and understand the image information captured from the camera, and to recognize and track the students’ body movements, such as fidgeting, nodding, and raising their hands.

The research [88] by Uzelac and Gligoric identified the voice characteristics of lecturers in the classroom environment, constructed a model using a random forest classifier, and established a link between the voice characteristics of lecturers and the feedback of students’ satisfaction. This enabled the real-time continuous prediction of students’ classroom satisfaction based on the voice characteristics, which in turn allowed teachers to adjust the speed of speech, interaction, and other lecturing styles in a timely manner according to the students’ satisfaction predicted by the model. The study compared the classification results with the distribution of students’ satisfaction with the segments on the webpage. The results demonstrated that the accuracy of this method in evaluating students’ satisfaction with the quality of lectures ranged from 70.7% to 83.9%.

In [89], a speech emotion recognition model was constructed for the purpose of analyzing the emotional information present in the teacher’s speech during the teaching process. This model was constructed using a hybrid neural network (HNN) as a classifier, with the specific aim of extracting three features: sound spectrogram, filter bank (FBank), and Mel-frequency cepstral coefficient (MFCC). DenseNet was employed as a convolutional neural network (CNN) module in the deep learning model training to convolve and merge the spectrogram image representation of the audio file to generate an expanded feature map. Subsequently, an LSTM architecture in the recurrent neural network (RNN) was utilized to process the sequence data and learn the feature vector sequence output by the CNN. Then, the distinct features processed by the CNN and LSTM were integrated through a parallel subnetwork to form a comprehensive feature vector, which was then transmitted to the fully connected layer and the Softmax layer for emotion classification. This process enables the recognition of emotions in speech. The lowest error rate that this method achieved was 24.64%.

As previously stated, facial expressions, voices, movements, and other signals are subjective expressions of students’ emotions. These expressions may exhibit certain deviations and may not necessarily represent the students’ true feelings. Conversely, measuring objective physiological features such as electroencephalography (EEG), heart rate, and body temperature should provide more reliable data. Abdulrahman et al. [44] proposed a method for emotion recognition based on EEG signals that employed deep learning. In this paper, the empirical mode decomposition (EMD) and variational mode decomposition (VMD) methods were employed to convert and preprocess EEG signals, simplify and decompose EEG signals into a series of modal functions (IMFs), and manually extract statistical features such as maximum, minimum, and average values from each IMF. The amplitude change of the signal was indicative of the intensity and stability of the students’ brain activity. Signal fluctuations of high amplitude and rapid changes indicate that students may be in a state of high enthusiasm and high participation, while vice versa, they may be in a calm state. Finally, the statistical features were input into the DeepBiLSTM deep learning model for feature classification. This method achieved an average accuracy of 70.89% in binary classification and an average accuracy of 90.33% in multivariate classification.

### 3.2. Data Analysis and Content Generation

In the previous subsection, we described the great potential of AI technology to help sensors understand information about the classroom environment. However, how AI technologies can help analyze previously understood data and output educational content is also of great interest. Data analysis and content generation algorithms do not directly process the data collected by the sensors, but rather empower the sensors with richer and more powerful features that enable them to play different roles in different teaching and learning sessions. For students, analytics and content generation algorithms can help achieve personalized learning and provide not only personalized, efficient teaching content support, but also the intelligent correction of students’ homework and the output of intelligent feedback; for educators, these algorithms help make educational decisions, predict the performance of students, automatically assess the quality of teaching, and reduce the pressure on the management of educators.

This subsection will demonstrate the application of AI technology in smart classrooms to facilitate the generation of decision-making and content software algorithms in three distinct areas: educational analytics and prediction, teaching quality assessment and feedback, and personalized learning and instructional support.

#### 3.2.1. Analysis and Prediction

The learning analysis capabilities of machine learning technology can be employed to achieve educational prediction and decision-making, including admission decisions, course scheduling, dropout and retention, and the prediction of academic performance. Commonly utilized analysis and prediction tools include logistic regression, traditional machine learning methods, and deep learning methods, among which machine learning methods exhibit superior classification accuracy compared to traditional logistic regression [7]. The following presents some representative studies.

The advent of online virtual education platforms has generated a vast quantity of educational data, which can be utilized to identify patterns in students’ learning behaviors and optimize educational decisions. Waheed et al. [90] employed a deep artificial neural network (Deep ANN) model to predict students’ academic performance from virtual learning environment (VLE) big data. The features extracted from VLE big data were manually completed. These features include the students’ highest education level, age, the click data of various activity types, delays in submitting homework, etc. Based on these features, the deep artificial neural network proposed in this paper can determine whether students can pass the course, whether they can pass the course with excellent results, and whether they will drop out of the course. The classification accuracy rate achieved was between 84% and 93%, which was better than the baseline logistic regression and support vector machine models in overall performance. This has the potential to enhance the educational decision-making process. In the study by Yagci [91], a variety of traditional machine learning methods were employed to predict the final exam scores of students enrolled in the Turkish course. The students’ midterm exam scores and faculty and department information were utilized as feature parameters to input into models constructed using machine learning algorithms, including Random Forests, Nearest Neighbor, Support Vector Machines, Logistic Regression, Naïve Bayes, and K-Nearest Neighbor. The efficiency differences between the methods were then compared. The classification accuracy of these machine learning algorithms ranged from 70% to 75%. The results of this study demonstrated that Random Forests, Neural Networks, and Support Vector Machines exhibited high predictive accuracy in predicting students’ academic performance and contributed to the early identification of students at high risk of failure.

Furthermore, machine learning algorithms have also been used to predict students’ job placement after graduation. Based on students’ academic performance in the tenth, twelfth, final year, and up to the graduation date, Maurya et al. [92] employed a variety of machine learning classifiers (such as support vector machines (SVM), Gaussian Naive Bayes, K-Nearest Neighbor, Stochastic Gradient Descent, Random Forest, Decision Tree, Logistic Regression, and Neural Network) to predict students’ positions in the IT industry. The best performing method, Stochastic Gradient Descent, achieved an accuracy of 91.17%.

#### 3.2.2. Teaching Quality Assessment and Student Feedback

In the past, the evaluation of teachers’ teaching quality was mostly based on observation, questionnaires, or grades. This process is subjective and may result in a low accuracy of the scoring system [93]. The integration of artificial intelligence technology is capable of enhancing the analysis of data collected by sensors to provide a more comprehensive and objective teaching evaluation. In addition, the application of AI technology can realize the automation of evaluation processes. By inputting data on satisfaction, classroom interaction, student test scores, homework submission, classroom participation, and other classroom process characteristics for training and classification, it can provide timely automated feedback and evaluation, and integrate it into learning activities to continuously analyze students’ performance, rather than stopping for testing to improve the efficiency of evaluation [94]. Lin [93] constructed an objective automatic teaching evaluation model with the weighted naive Bayes algorithm. This modal was able to enhance the efficiency and performance of the evaluation model when applied to a larger scale of teaching evaluation data. The discrepancy between the model output and the standard manual score was no greater than 10%, which rendered the model capable of replacing manual scoring. To enhance the precision of the teaching evaluation model and mitigate the impact of various confounding variables in the evaluation process, Sun [95] employed the ACLLMD method (a signal processing method) to decompose and eliminate power quality interference signals, resolving issues encountered in the signal decomposition process and enhancing the Relevance Vector Machine (RVM) machine learning algorithm in the feature extraction stage. Moreover, Huang [96] proposed an active learning algorithm based on a hybrid Gaussian process and an improved correlation vector machine model. This algorithm improved the efficiency and accuracy of ELT assessment by strategically selecting and labeling samples.

The assessment of student learning outcomes represents a crucial responsibility for educators at all levels of academic instruction. With regard to written assignments, it is undoubtedly one of the most challenging, laborious, and time-consuming tasks [97]. In 2014, a system for automatically grading computer programs using machine learning was first proposed in [98].

In general, student work is divided into closed and open questions. Closed question correction is easy to grade; as these questions have a single limited number of correct answers, the general way to achieve grading is to compare the similarity of the answers with the reference answers, and there are already efficient correction procedures. However, for open-ended subjective short-answer questions, there are often no standard answers for these questions, which require a great deal of teacher attention and are easily influenced by the subjectivity of the grader. Automatic machine grading will effectively improve this problem, and the progress of deep learning algorithms is expected to promote research in this field. Zhang et al. [99] developed an automatic semi-open-ended short answer scoring model. The paper employed a long short-term memory recurrent neural network (LSTM) to learn the representation in the classifier, which enabled the model to consider word sequence information. The author integrated general domain information from Wikipedia and specific domain information from labeled student answers to train the model, and set up an experiment with seven reading comprehension questions and more than 16,000 short answer questions. The results showed that the automatic scoring model was superior to the existing model. However, this study also suffered from the typical issues of using deep neural network technology, which required a large amount of labeled data for training. Moreover, once trained, it was equivalent to being locked and could not dynamically adapt to changes in the environment, resulting in a decline in evaluation performance. Jamil and Hameed [100] constructed a real-time ISE (Intelligent Student Evaluation) system based on DNN and NLP techniques that dynamically evaluates students’ answers. The model used Particle Swarm Optimization (PSO) and Gradient Descent (GD) as optimization schemes for model weight parameters, which allowed the model to adjust itself when encountering new data, thus enabling continuous learning to adapt to new data and environmental changes. The correction of math problems is also a typical application scenario for open-ended questions. Botelho et al. [101] developed a system for automatically assessing students’ open-ended math questions using Natural Language Processing (NLP) techniques. It was mainly a model based on machine learning and collaborative filtering methods, and this system was able to give feedback and recommendations in addition to scores.

#### 3.2.3. Personalized Learning and Instructional Support

According to the 2017 U.S. National Education Technology Plan, personalized learning is defined as “instruction that optimizes the pace of learning and teaching methods based on the needs of each learner” [102]. Ezzaim et al. [103] proposed a definition of adaptive learning: the notion of adaptive learning can be defined as a technology-based approach represented by educational systems and platforms that try to tailor pedagogical content, presentation styles, or learning paths to individual profiles, such as cognitive state, affective status, and knowledge level. In academia, the two terms personalized learning and adaptive learning are used interchangeably [104]. Personalized learning has existed for hundreds of years in the form of apprenticeship and mentoring, and as educational technology began to mature in the second half of the last century, personalized learning emerged in the form of intelligent tutoring systems.

In this century, advances in big data and learning analytics are expected to transform personalized learning once again [105], especially represented by the great potential that AI technologies hold in the field of personalized learning. The realization of personalized learning requires machines to be able to analyze and understand the personal characteristics of different learners. AI-based processing algorithms are capable of capturing educational data from sensors or online system backgrounds, and output personalized content to provide instructional support.

In large-scale distance learning institutions, AI can fully exert its advantages. These institutions run modules for thousands of students, providing a rich learning database for training artificial intelligence to generate personalized learning paths, because educators can collect a large amount of students’ learning interaction data in the education platform including click-through rate, learning time, problem-solving speed, and facial expressions. These data do not come from traditional sensors, but from the background of the program system. In this setting, machine learning technology which is capable of processing large amounts of data is more efficient than traditional algorithms. Machine learning technology can learn and analyze students’ learning behavior patterns from a large amount of data. Subsequently, compared with traditional e-learning systems that provide similar content to all learners, machine learning-based systems can provide specific learning routes that suit the needs of each learner. In addition, the application learning system of natural language processing (NLP) technology and emotion recognition can understand learners’ emotions and expressions through sensors or language input, and combined with content generation technology, AI-based intelligent learning systems can play a role as a simulated teacher, providing learners with real-time automatic one-to-one targeted learning feedback and guidance without the need for a large number of teachers online. Furthermore, this system is able to continuously track the real-time interaction of remote students and adapt to the long-term growth and changes of learners.

A growing number of studies have been conducted employing artificial intelligence (AI) algorithms to address challenges in personalized learning. Abyaa et al. [106] developed an automatic classifier based on a supervised learning algorithm that can predict learners’ personality dimensions based on their learning traces in online learning systems, which is very important in the design of personalized learning paths.

New learners to adaptive learning systems may encounter a “cold start problem”, which is, the system usually has no information about the initial ability level of new learners entering the learning environment. Therefore, it is challenging to accurately predict the proficiency of these new learners, which may impact the quality of personalized item recommendations in the initial stage of the learning environment. In order to improve the adaptability of the system, Pliakos et al. [107] proposed a system that combines item response theory (IRT) with machine learning. The new learner’s side information (including age, relevant courses taken, IQ, and pre-test scores) and responses were used as machine learning training sets. When new learners enter the system, the trained machine learning model is used to predict their potential ability parameters based on their background information. The predicted ability parameters are then combined with the IRT model to predict the new learner’s response to learning items. This enabled the system to provide more personalized learning materials while only having the most basic side information of the learner, alleviating the impact of the cold start problem. This article also mentioned that the IRT model combined with random forest provided the lowest error and highest response prediction accuracy in ability estimation.

Additionally, adaptive learning platforms based on AI technology can also provide personalized support to educators. In the process of designing online courses, educators often dedicate significant time and effort to retrieving learning objects (LOs) to develop suitable courses. Tahir et al. [108] proposed an intelligent system called DRFLO (Dynamic Recommendation of Filtered Learning Objects) based on machine learning technology and context-based recommendation methods. This system was designed to assist course designers in searching and accessing high-quality learning resources that aligned with their teaching objectives and course design based on their preferences and the current progress of the teaching context.

Advances in natural language processing and emotion recognition technologies can also provide learners with personalized learning support in the form of educational robots. By integrating AI-powered chatbots (based on the Amazon Lex platform) into e-learning, the system proposed in [109] by Davies et al. was able to provide customized learning material based on different user parameters and make up for the lack of real-time consultation in offline courses. However, at present, the level of chatbots cannot replace real educators and can only serve as online assistants.

Lu et al. [110], based on the self-determination theory (SDT), designed a physical learning assistant robot SLP (Smart Learning Partner) for middle school students. This system supported informal chats with students through a conversational agent engine to enhance students’ sense of social connection, and provided real-time feedback through emotion recognition technology, which adapted to students’ emotional states. In addition, the system can also utilize personal assessment results and interaction data with the question-and-answer engine to automatically label different levels of knowledge mastery for each concept, provide corresponding multidisciplinary learning materials, and achieve a personalized learning experience. When students demonstrate significant progress in the current learning topic, the SLP can also provide regular positive feedback and encouragement, and encourage students to attempt more challenging learning topics to enhance students’ sense of challenge and achievement. This system was a powerful assistant for learners for social interactions and daily learning activities.

Educational robot systems can also provide personalized support for specific learning content. Jiao et al. [111] proposed an English oral teaching system based on speech recognition and machine learning. Through the deep belief network (DBN) support vector machine (SVM) model, the pronunciation errors of oral learners were classified and detected, and the quality can be intelligently scored and pronunciation errors can be corrected. This system served as a personal oral learning partner and provided a new English oral teaching model. Similarly, Liu et al. [112] employed an intelligent dialogue robot to practice drama dialogue with students, thereby helping them to learn English speaking. This intelligent oral learning system, called SmartVpen, integrated the context-aware intelligent learning mechanism (CASLM) to perceive the learner’s learning content and provide oral feedback, enabling learners to practice drama dialogue independently.

### 3.3. Summary of AI Integrated Sensor Software Algorithms in Classroom

This section reviews the combination of AI technology and sensor software algorithms from the perspectives of recognition algorithms, analysis and prediction, and content generation algorithms, demonstrating its broad application prospects. Artificial intelligence technology has significantly improved the efficiency and accuracy of data processing and analysis. Machine learning technology has played its advantages in sensor data regression and cluster analysis, and has achieved considerable recognition accuracy in image recognition, speech recognition, EEG signal analysis, and other tasks, enhancing the monitoring and understanding of student behavior and emotions. In particular, the development of deep neural network technology (DNN) has brought progress and huge development potential to recognition algorithms. Deep learning algorithms represented by convolutional neural networks (CNN) are particularly suitable for processing large amounts of image and video data. This algorithm does not require manual feature extraction and has better accuracy. However, deep learning technology has recognition limitations or decreased accuracy for data with less data volume, more details, and fewer classification dimensions, such as micro-expressions. In some studies, these algorithms are often improved or combined with other machine learning algorithms to adapt to the characteristics of different recognition scenarios and achieve better results. Additionally, after the epidemic, the number of studies on data recognition algorithms for online learning students based on sensor devices that are limited by remote online learning (often cameras and microphones built into personal computers or smartphones) has also increased.

Artificial intelligence technology expanded the scope of sensors. While machine learning technology helps improve recognition efficiency, it can also learn the underlying patterns within data and analyze students’ learning behavior data to help predict academic success or recommend personalized learning paths. Based on AI technology, sensors can also serve as “teaching supervisors” and “grading assistants”, evaluating teachers’ pedagogical quality in real time in class, grading homework after class (auto-grading), obtaining students’ learning status, and obtaining objective feedback on teaching quality. Natural language processing technology (NLP) demonstrated outstanding performance in speech recognition and sentiment analysis. In certain studies, it has also been shown to be effective when combined with large language models and voice or display output devices, enabling it to act as an intelligent tutor chatbot, establish one-on-one communication with students, and replace teachers to complete certain teaching and question-answering tasks.

## 4. Discussion

The use of artificial intelligence systems inevitably entails the collection of vast quantities of data, including confidential information about students and teachers, which raises serious privacy and data protection issues [7]. Only two (1.4%) of the 146 articles retrieved in the survey in [7] in 2019 critically reflect on the ethical implications, challenges, and risks of AI applications in education.

In some educational classrooms, cameras are still installed in classrooms in a way that is both conspicuous and intrusive to each students’ desk, though in experimental scenarios, which may still give students the psychological burden of being watched, raise privacy concerns, and may lead to a decrease in classroom efficiency. Moreover, several faculty, teaching assistants, student counselors, and administrators may be concerned that intelligent tutoring systems, expert systems, and chatbots based on artificial intelligence technology will take away their jobs. These are ethical issues that cannot be ignored. We call on more researchers in the future to invest in research on data privacy and ethical issues brought about by the introduction of emerging sensor devices in smart classrooms, help protect the basic rights and interests of students and educators, and enhance the acceptance of sensor technology at the use and social levels.

Usability is another significant challenge. The term “usability” is used to describe the effectiveness, efficiency, and subjective satisfaction of a product when the user uses it. Although numerous new sensor devices have been introduced in smart classrooms, these devices still have problems with comfort and invasiveness. Some of these devices are directly and conspicuously exposed in the classroom environment, while others, such as wearable monitoring devices, may cause a poor wearing experience due to weight and volume. Such situations will result in physical and psychological rejection and a sense of unfamiliarity among students and teachers, thereby affecting the effectiveness of teaching. In order to improve the usability of sensor devices, future research may be directed towards reducing the visual presence of sensor devices in educational settings, hiding the devices in corners, or developing more comfortable wearable devices. The interface of the sensor involves viewing data information. How to present data information in a clearer and more visual way also represents an important area for usability enhancement, which involves the knowledge of human–computer interfaces and information design.

Since the pandemic, the trend of “remote” and “virtual” education has become increasingly prominent, with the potential for a radical transformation of the teaching scenario. The question of how to achieve a mastery of the classroom situation in a remote online classroom or a classroom in a virtual reality space, and how to design new sensor systems are issues that are rarely discussed. In the virtualized world, sensors may exist in new forms. It is conceivable that sensor systems may be represented by a string of code that calls background data, or integrated into wearable virtual reality devices.

## 5. Conclusions

The integration of sensor technology and artificial intelligence in smart classrooms is constantly innovating the current education methods, greatly enhancing the “perception” ability of the classroom, and providing strong support for interactive, personalized, and intelligent teaching. This review introduces the application of various sensor technologies in smart classrooms and their deep integration with AI algorithms, and summarizes the main trends and challenges of current technology applications.

The sensor system can play multiple roles in a smart classroom. It monitors the physical conditions of the classroom in real time and is committed to creating the most comfortable and efficient teaching environment. The sensor system can also capture subtle facial emotions, body movements, sounds, brain waves, and other signals from a professional and objective perspective to evaluate the status of teachers and students during the teaching process, serving as a teaching supervisor. Sensor systems deployed with artificial intelligence technology perform a wider range of functions. Such systems can act as one-to-one intelligent tutors, learning from data, analyzing, and summarizing students’ learning behavior characteristics. They can also output academic prediction reports, promote personalized learning plans and content, provide educational chat exchanges, and provide comprehensive intelligent educational support.

Sensor systems significantly improve teaching in smart classrooms; however, they also require a high deployment cost. The development of low-cost and affordable sensor solutions is urgently needed. Deep learning technology provides some more efficient and accurate recognition solutions, but it requires large-scale datasets, massive computing resources and a large amount of memory, and the training and testing phases are very time-consuming, which is challenging for some educational organizations with limited funds and resources to provide independently. The cost of some advanced biosensor devices that can provide objective physiological data is also a significant barrier to the promotion of sensor technology. Yet, few studies have focused on reducing the deployment cost of deep learning algorithms, and further research is necessary. One potential avenue for future research is an investigation of the built-in sensors of personal mobile devices, such as cameras integrated into smartphones or tablet devices. In numerous classrooms, sensors on personal devices have been shown to complete attendance tasks at a low cost. These devices have been employed for activities such as QR code scanning check-in, geolocation check-in, and identity recognition. They do not necessitate additional equipment configuration costs and can be utilized in sufficient quantities. In addition, they are more accessible and user-friendly, and generally require the development of a mobile application to invoke them. However, there are few studies investigating the utilization of such sensors to complete other educational applications, such as participation monitoring and personalized learning. As the reform of smart classrooms in education and teaching emphasizes intelligence while paying attention to cost-effectiveness, the balance between the cost of intelligent systems in teaching classrooms and teaching gains is a very delicate issue that deserves in-depth exploration.

This work also identified several trends in the application of sensors in smart classrooms. The use of wearable sensor devices and personal smart mobile devices has been increasingly prevalent in smart classroom settings. Sensor devices are developing towards miniaturization, integration, and wearability. Furthermore, with the advancement of virtual reality technology and remote teaching technology, new types of sensor devices for remote virtual teaching spaces will emerge to adapt to the “virtualization” of classrooms. Of course, these trends are inseparable from the synchronous development of artificial intelligence technology. The advent of deep learning technology and large language model technology have shown their immense potential to help further improve the application effect of sensors at the software algorithm level.

Nevertheless, the application of smart classroom technology still encounters challenges including data privacy and security, deployment costs, availability, and new application settings. Future research should focus on solving these issues and developing safer, more efficient, and more usable technologies and algorithms. At the same time, more exploration of multi-sensor integration, sensor data personalized learning, and intelligent recommendation should be conducted, which can provide novel insights for the comprehensive promotion and application of smart classrooms.

Yet, the integration of sensors and AI technology in smart classrooms has brought unprecedented opportunities and challenges to the intelligentization of education. Through continued technological innovation and application research, smart classrooms will become the main model of future education, helping to promote the intelligent, personalized and interactive development of education, and providing teachers and students with enhanced teaching and learning experiences.

## Figures and Tables

**Table 1:** Environmental sensors in smart classrooms.

Sensor Type	Monitored Feature	Monitoring Purpose	Typical Studies
Thermal sensor	Temperature	Classroom climate temperature	[10]
Acoustic sensor	Sound level meter	Detecting noise levels	[11,12]
Infrared radio sensor	Infrared energy	Scouting for student activity in the classroom	[13]
Carbon dioxide sensors	Carbon dioxide content	Monitoring of carbon dioxide levels in the air	[10,14]
Photosensitive sensor	Lighting conditions	Monitoring light levels and uniformity	[15,16]
Infrared camera	Infrared image	Student body temperature	[17]

**Table 2:** Image-based biometric sensors for engagement analysis.

Sensor Type	Monitored Feature	Monitoring Purpose	Typical Studies
Facial recognition system	Facial features	Identification and emotional state analysis	[25]
Posture and motion sensor	Body posture	Engagement analysis and classroom dynamics	[26]
Thermal camera	Body temperature	Health monitoring and stress analysis	[17]
Eye-tracking device	Eye movement and pupil dilation	Focus and engagement analysis	[27,28,29,30]

**Table 4:** Biometric sensors for attendance.

Sensor Type	Monitored Data	Monitoring Purpose	Typical Studies
Camera	Facial or body image features	Facial features identification	[50]
Fingerprint Sensor	Fingerprint patterns	Fingerprint features identification	[49]
Acoustic Sensors	Voice characteristics	Voice features identification	[52]
Pressure Sensor	The pressure applied to the chair	Detect if someone is sitting in the chair	[53]
Infrared Camera	Infrared motion image	Detect movements	[54]

**Table 5:** Image-based recognition algorithms integrated with AI technology.

Types of Recognition Sensors	Recognized Features	Recognition Purpose	Classifier	Accuracy	Typical Study
Computer camera	Student facial images	Emotion classification	SVM Regression	99.16%	[68]
Classroom wall-mounted camera	Student facial images	Dynamically evaluate classroom performance	CNN	70.1%	[69]
Classroom wall-mounted camera	Student facial images	Automated attendance	CNN	99%	[70]
Classroom wall-mounted camera	Student facial images	Facial feature recognition, emotion recognition, classroom sign-in	MTCNN	Facial recognition: 98% Emotion recognition: 92%	[71]
Classroom wall-mounted camera	Student facial images	Student identification	KNN	93.94%	[72]
Classroom wall-mounted camera	Student facial images	Face recognition under blurry conditions	YOLOv5	81.42%	[73]
Computer camera	Student facial images	Confused emotion recognition	CNN–SVM	93.8%	[74]
Computer camera	Student body movement images	Student action recognition	CNN	89.5%	[75]

**Table 6:** Non-image-based recognition algorithms integrated with AI technology.

Types of Recognition Sensors	Recognized Features	Recognition Purpose	Classifier	Accuracy	Typical Study
Acoustic Sensor	Student Voice	Understanding student interest levels	Adaboost M1	81.9%	[37]
Acoustic Sensor	Teacher’s Voice	Reflecting student satisfaction	Random Forest	70.7%~83.9%	[88]
Acoustic Sensor	Teacher’s Voice	Identify emotional information in teachers’ voice during teaching	CNN and LSTM	75.36%	[89]
EEG Sensors	Student EEG signals	Identifying student emotions	DeepBi LSTM	Binary classification: 70.89% Multivariate classification: 90.33%	[44]

## Data Availability

No new data were created or analyzed in this study. Data sharing is not applicable to this article.

## References

[1] Roberts J. (2000). From know-how to show-how? Questioning the role of information and communication technologies in knowledge transfer. Technol. Anal. Strateg. Manag..

[2] Saini M.K., Goel N. (2020). How Smart Are Smart Classrooms? A Review of Smart Classroom Technologies. ACM Comput. Surv..

[3] Dimitriadou E., Lanitis A. (2023). A critical evaluation, challenges, and future perspectives of using artificial intelligence and emerging technologies in smart classrooms. Smart Learn. Environ..

[4] Spikol D., Ruffaldi E., Dabisias G., Cukurova M. (2018). Supervised machine learning in multimodal learning analytics for estimating success in project-based learning. J. Comput. Assist. Learn..

[5] Alfoudari A.M., Durugbo C.M., Aldhmour F.M. (2021). Understanding socio-technological challenges of smart classrooms using a systematic review. Comput. Educ..

[6] Wang Y.H., Lu S.L., Harter D. (2021). Multi-Sensor Eye-Tracking Systems and Tools for Capturing Student Attention and Understanding Engagement in Learning: A Review. IEEE Sens. J..

[7] Zawacki-Richter O., Marin V.I., Bond M., Gouverneur F. (2019). Systematic review of research on artificial intelligence applications in higher education—Where are the educators?. Int. J. Educ. Technol. High. Educ..

[8] Hu Y., Huang R. Development of Weather Monitoring System based on Raspberry Pi for Technology Rich Classroom. Proceedings of the International Conference on Smart Learning Environments (ICSLE).

[9] Mendell M.J., Heath G.A. (2005). Do indoor pollutants and thermal conditions in schools influence student performance? A critical review of the literature. Indoor Air.

[10] Stazi F., Naspi F., Ulpiani G., Di Perna C. (2017). Indoor air quality and thermal comfort optimization in classrooms developing an automatic system for windows opening and closing. Energy Build..

[11] Marques G., Pitarma R. (2020). A Real-Time Noise Monitoring System Based on Internet of Things for Enhanced Acoustic Comfort and Occupational Health. IEEE Access.

[12] de Valencia A.C., Caballero L., Cilli A., González J., Rojas I., Torres B. Development of a noise monitoring and control sensor network system for the enclosed spaces within a university environment. Proceedings of the IEEE Latin-American Conference on Communications (LATINCOM).

[13] Twumasi C., Dotche K.A., Banuenumah W., Sekyere F. Energy Saving System using a PIR Sensor for Classroom Monitoring. Proceedings of the IEEE PES-IAS PowerAfrica Conference.

[14] Pastor F.J.F., Horcajadas M.P., Davo J.A.L., Iglesias V.G. Developing Environmental Adaptative Comfort Using Internet of Things and Business Process Management: Application in a University Building. Proceedings of the International Conference on Ubiquitous Computing & Ambient Intelligence (UCAmI).

[15] Amelkina S.A., Duplenkova K.A. (2021). Justification Lighting Control System Using in the Classes on the Basis of Lighting Scene Simulation. Light Eng..

[16] Chen J.B., Xue R., Liu P.X., Zhang X. Design of Sub-regional Lighting Control System for Classroom. Proceedings of the 7th International Conference on Applied Science, Engineering and Technology (ICASET).

[17] Alkhayat A.H., Bagheri N., Ayub M.N., Noor N.F.M. Fever Detection & Classroom Temperature Adjustment Using Infrared Cameras. Proceedings of the IEEE International Conference on Consumer Electronics—Taiwan (ICCE-TW 2015).

[18] Chiou C.K., Tseng J.C.R. An Intelligent Classroom Management System based on Wireless Sensor Networks. Proceedings of the 8th International Conference on Ubi-Media Computing (UMEDIA).

[19] Deepaisarn S., Angkoonsawaengsuk A., Arunkit C., Srisumarnk C., Nimmanwatthana K., Linphrachaya N., Chiewnawintawat N., Tanthanathewin R., Seinglek S., Buaruk S. Camera-Based Log System for Human Physical Distance Tracking in Classroom. Proceedings of the 14th Annual Summit and Conference of the Asia-Pacific-Signal-and-Information-Processing-Association (APSIPA ASC).

[20] Du X., Zhao S., Zhang D., Yu Y. (2023). Comparative analysis of light environment perception, eye movement and physiology in university professional classroom based on virtual reality experiment. Indoor Built Environ..

[21] Zola Cruz I., Garizurieta Bernabe J., Gonzalez Benitez R.A., Morales Toxqui J. Design of a prototype for automatic lighting control in classrooms at Faculty of Accounting and Administration Xalapa based on the IoT paradigm. Proceedings of the IEEE International Conference on Engineering Veracruz (IEEE ICEV).

[22] Peifer C., Engeser S. (2021). Advances in Flow Research.

[23] Carroll M., Lindsey S., Chaparro M., Winslow B. (2021). An applied model of learner engagement and strategies for increasing learner engagement in the modern educational environment. Interact. Learn. Environ..

[24] Verner C., Dickinson G. (1967). The lecture, an analysis and review of research. Sage J..

[25] Alkabbany I., Ali A.M., Foreman C., Tretter T., Hindy N., Farag A. (2023). An Experimental Platform for Real-Time Students Engagement Measurements from Video in STEM Classrooms. Sensors.

[26] Zhu Y.J., Wang S., Peng Y., Chen Y.Q., Chen R.L., Ke G., Li J.X. (2023). Research and Implementation of Intelligent Learning Desk Based on Visio Sensor in AI IoT Environments for Smart Education. Sens. Mater..

[27] MacInnes J.J., Iqbal S., Pearson J., Johnson E.N. (2018). Wearable Eye-tracking for Research: Automated dynamic gaze mapping and accuracy/precision comparisons across devices. bioRxiv.

[28] Hu R., Hui Z., Li Y., Guan J. (2023). Research on Learning Concentration Recognition with Multi-Modal Features in Virtual Reality Environments. Sustainability.

[29] Kassner M., Patera W., Bulling A. Pupil: An Open Source Platform for Pervasive Eye Tracking and Mobile Gaze-based Interaction. Proceedings of the ACM International Joint Conference on Pervasive and Ubiquitous Computing (UbiComp).

[30] Shvarts A., Abrahamson D. (2019). Dual-eye-tracking Vygotsky: A microgenetic account of a teaching/learning collaboration in an embodied-interaction technological tutorial for mathematics. Learn. Cult. Soc. Interact..

[31] Kim P.W. (2019). Ambient intelligence in a smart classroom for assessing students’ engagement levels. J. Ambient Intell. Humaniz. Comput..

[32] Alemdag E., Cagiltay K. (2018). A systematic review of eye tracking research on multimedia learning. Comput. Educ..

[33] Zaletelj J. Estimation of Students’ Attention in the Classroom From Kinect Features. Proceedings of the 10th International Symposium on Image and Signal Processing and Analysis.

[34] Prieto L.P., Sharma K., Kidzinski L., Rodríguez-Triana M.J., Dillenbourg P. (2018). Multimodal teaching analytics: Automated extraction of orchestration graphs from wearable sensor data. J. Comput. Assist. Learn..

[35] Gunawardena N., Ginige J.A., Javadi B. (2023). Eye-tracking Technologies in Mobile Devices Using Edge Computing: A Systematic Review. ACM Comput. Surv..

[36] Jarodzka H., Skuballa I., Gruber H. (2021). Eye-Tracking in Educational Practice: Investigating Visual Perception Underlying Teaching and Learning in the Classroom. Educ. Psychol. Rev..

[37] Gligoric N., Uzelac A., Krco S., Kovacevic I., Nikodijevic A. (2015). Smart classroom system for detecting level of interest a lecture creates in a classroom. J. Ambient Intell. Smart Environ..

[38] Basu S. (2002). Conversational Scene Analysis. Ph.D. Thesis.

[39] Chen C.M., Wang J.Y., Lin M. (2019). Enhancement of English learning performance by using an attention-based diagnosing and review mechanism in paper-based learning context with digital pen support. Univers. Access Inf. Soc..

[40] Carroll M., Ruble M., Dranias M., Rebensky S., Chaparro M., Chiang J., Winslow B. (2020). Automatic detection of learner engagement using machine learning and wearable sensors. J. Behav. Brain Sci..

[41] Lascio E.D., Gashi S., Santini S. (2018). Unobtrusive Assessment of Students’ Emotional Engagement during Lectures Using Electrodermal Activity Sensors. Proc. ACM Interact. Mob. Wearable Ubiquitous Technol..

[42] Romine W.L., Schroeder N.L., Graft J., Yang F., Sadeghi R., Zabihimayvan M., Kadariya D., Banerjee T. (2020). Using Machine Learning to Train a Wearable Device for Measuring Students’ Cognitive Load during Problem-Solving Activities Based on Electrodermal Activity, Body Temperature, and Heart Rate: Development of a Cognitive Load Tracker for Both Personal and Classroom Use. Sensors.

[43] Hsu C.C., Chen H.C., Su Y.N., Huang K.K., Huang Y.M. (2012). Developing a Reading Concentration Monitoring System by Applying an Artificial Bee Colony Algorithm to E-Books in an Intelligent Classroom. Sensors.

[44] Abdulrahman A., Baykara M., Alakus T.B. (2022). A Novel Approach for Emotion Recognition Based on EEG Signal Using Deep Learning. Appl. Sci..

[45] Dias B., Mohammad A., Xu H., Tan P. Intelligent Student Attendance Management System Based on RFID Technology. Proceedings of the 13th International Conference on Complex, Intelligent, and Software Intensive Systems (CISIS).

[46] Fernández M.J.L., Fernández J.G., Aguilar S.R., Selvi B.S., Crespo R.G. (2013). Control of attendance applied in higher education through mobile NFC technologies. Expert Syst. Appl..

[47] Zoric B., Dudjak M., Bajer D., Martinovic G. Design and development of a smart attendance management system with Bluetooth low energy beacons. Proceedings of the Zooming Innovation in Consumer Technologies Conference (ZINC).

[48] Banepali A., Kadel R., Guruge D.B., Halder S.J. Design and Implementation of Wi-Fi Based Attendance System Using Raspberry Pi. Proceedings of the 29th International Telecommunication Networks and Applications Conference (ITNAC).

[49] Gagandeep, Arora J., Kumar R. (2019). Biometric fingerprint attendance system: An internet of things application. Innovations in Computer Science and Engineering, Proceedings of the Innovations in Computer Science and Engineering: Proceedings of the Fifth ICICSE 2017, Hyderabad, India, 18–19 August 2017.

[50] Ni L., Shi J., Han B., Zhang N., Lan Q.M., Su Z.H. Classroom Roll Call System Based on Face Detection Technology. Proceedings of the 10th International Conference on Information and Education Technology (ICIET).

[51] Sweena K.S., Lisa M.R., Shiny P.C., Sancy S.P., Paul J.J., Thusnavis B.M.I. Chatbot—Attendance and Location Guidance system (ALGs). Proceedings of the 3rd International Conference on Signal Processing and Communication (ICPSC).

[52] Amri U.F., Hashim N.N.W.N., Hanif N.H.H.M. Speech-based Class Attendance. Proceedings of the 6th International Conference on Mechatronics (ICOM), Int Islam Univ Malaysia, Gombak Campus.

[53] He J., Atabekov A., Haddad H.M. Internet-of-Things Based Smart Resource Management System: A Case Study Intelligent Chair System. Proceedings of the 25th IEEE International Conference on Computer Communications and Networks (ICCCN).

[54] Yadav M., Aggarwal A., Rakesh N. Motion Based Attendence System In Real-Time Environment for Multimedia Application. Proceedings of the 8th IEEE International Conference on Cloud Computing, Data Science and Engineering (Confluence)/Global Technology, Innovation and Enterpreneurship Summit, Amity Sch Engn & Technol.

[55] Rahmatya M.D., Wicaksono M.F. Design of Student Attendance Information System with Fingerprints. Proceedings of the 2nd International Conference on Informatics, Engineering, Science, and Technology (INCITEST)—Building Competitive Advantage to Face Industry 4.0.

[56] Adal H., Promy N., Srabanti S., Rahman M. Android Based Advanced Attendance Vigilance System Using Wireless Network with Fusion of Bio-metric Fingerprint Authentication. Proceedings of the 20th International Conference on Advanced Communication Technology (ICACT).

[57] Sweetlin J.D., Aswini V., Dhanusha R. Speech Based Attendance Application Register. Proceedings of the 5th International Conference on Recent Trends in Information Technology (ICRTIT).

[58] Hoo S.C., Ibrahim H. (2019). Biometric-Based Attendance Tracking System for Education Sectors: A Literature Survey on Hardware Requirements. J. Sens..

[59] Sarker D.K., Hossain N.I., Jamil I.A. Design and Implementation of Smart Attendance Management System Using Multiple Step Authentication. Proceedings of the International Workshop on Computational Intelligence (IWCI).

[60] Veer N.D., Momin B.F. An automated attendance system using video surveillance camera. Proceedings of the IEEE International Conference on Recent Trends in Electronics, Information and Communication Technology (RTEICT).

[61] Varshini M.R.L., Vidhyapathi C.M. Dynamic Fingure Gesture Recognition using KINECT. Proceedings of the International Conference on Advanced Communication Control and Computing Technologies (ICACCCT).

[62] Zhang X.Y., Kulkarni H., Morris M.R. Smartphone-Based Gaze Gesture Communication for People with Motor Disabilities. Proceedings of the ACM SIGCHI Conference on Human Factors in Computing Systems (CHI).

[63] Lathière B., Archambault D. Improving Accessibility of Lectures for Deaf and Hard-of-Hearing Students Using a Speech Recognition System and a Real-Time Collaborative Editor. Proceedings of the 14th International Conference on Computers Helping People with Special Needs (ICCHP).

[64] Hornof A.J., Cavender A. EyeDraw: Enabling children with severe motor impairments to draw with their eyes. Proceedings of the SIGCHI Conference on Human Factors in Computing Systems.

[65] Baker T., Smith L. (2019). Educ-AI-Tion Rebooted? Exploring the Future of Artificial Intelligence in Schools and Colleges. https://www.nesta.org.uk/report/education-rebooted/.

[66] Silva L.C.E., Sobrinho A., Cordeiro T.D., Melo R.F., Bittencourt I.I., Marques L.B., Matos D., da Silva A.P., Isotani S. (2023). Applications of convolutional neural networks in education: A systematic literature review. Expert Syst. Appl..

[67] Ko B.C. (2018). A Brief Review of Facial Emotion Recognition Based on Visual Information. Sensors.

[68] Sabri N., Musa N.H., Mangshor N.N.A., Ibrahim S., Hamzah H.H.M. (2020). Student emotion estimation based on facial application in E-learning during COVID-19 pandemic. Int. J. Adv. Trends Comput. Sci. Eng..

[69] Tang J.L., Zhou X.T., Zheng J.W. Design of Intelligent classroom facial recognition based on Deep Learning. Proceedings of the International Conference on Computer Information Science and Application Technology (CISAT).

[70] Filippidou F.P., Papakostas G.A. Single Sample Face Recognition Using Convolutional Neural Networks for Automated Attendance Systems. Proceedings of the 4th International Conference on Intelligent Computing in Data Sciences (ICDS).

[71] Wang Y.H., Choo W.O., Wang X.F. (2023). Face Recognition Technology Based on Deep Learning Algorithm for Smart Classroom Usage. J. Eng. Sci. Technol..

[72] Hu M.J., Wei Y.T., Li M.S.Y., Yao H., Deng W., Tong M.W., Liu Q.T. (2022). Bimodal Learning Engagement Recognition from Videos in the Classroom. Sensors.

[73] Bie M., Liu Q.L., Xu H., Gao Y., Che X.J. (2023). FEMFER: Feature enhancement for multi-faces expression recognition in classroom images. Multimed. Tools Appl..

[74] Shi Z., Zhang Y., Bian C.L., Lu W.G. Automatic Academic Confusion Recognition In Online Learning Based On Facial Expressions. Proceedings of the 14th International Conference on Computer Science and Education (ICCSE).

[75] Qi Y., Zhuang L., Chen H., Han X., Liang A. (2024). Evaluation of Students’ Learning Engagement in Online Classes Based on Multimodal Vision Perspective. Electronics.

[76] Tarnowski P., Kołodziej M., Majkowski A., Rak R.J. (2017). Emotion recognition using facial expressions. Procedia Comput. Sci..

[77] Lek J.X.Y., Teo J. (2023). Academic Emotion Classification Using FER: A Systematic Review. Hum. Behav. Emerg. Technol..

[78] Chiu C.K., Tseng J.C.R. (2021). A Bayesian Classification Network-based Learning Status Management System in an Intelligent Classroom. Educ. Technol. Soc..

[79] Jadhav R.S., Ghadekar P. Content Based Facial Emotion Recognition Model using Machine Learning Algorithm. Proceedings of the International Conference on Advanced Computation and Telecommunication (ICACAT).

[80] Lasri I., Solh A.R., El Belkacemi M. Facial Emotion Recognition of Students using Convolutional Neural Network. Proceedings of the 3rd International Conference on Intelligent Computing in Data Sciences (ICDS).

[81] Gao Z., Huang Y., Zheng L., Li X., Lu H., Zhang J., Zhao Q., Diao W., Fang Q., Fang J. (2021). A student attendance management method based on crowdsensing in classroom environment. IEEE Access.

[82] Wu H., Pan Y., Weng X., Chen H. Design of Campus Health Information System Using Face Recognition and Body Temperature Detection. Proceedings of the 2021 IEEE Intl Conf on Dependable, Autonomic and Secure Computing, Intl Conf on Pervasive Intelligence and Computing, Intl Conf on Cloud and Big Data Computing, Intl Conf on Cyber Science and Technology Congress (DASC/PiCom/CBDCom/CyberSciTech).

[83] Li J., Jin K., Zhou D., Kubota N., Ju Z. (2020). Attention mechanism-based CNN for facial expression recognition. Neurocomputing.

[84] Guo Y.F., Huang J., Xiong M.F., Wang Z.Y., Hu X.R., Wang J.H., Hijji M. (2022). Facial expressions recognition with multi-region divided attention networks for smart education cloud applications. Neurocomputing.

[85] Zhang X.L., Cao Z.Q. (2021). A Framework of an Intelligent Education System for Higher Education Based on Deep Learning. Int. J. Emerg. Technol. Learn..

[86] Rao K.P., Chandra M.V.P., Rao S. (2020). Recognition of learners’ cognitive states using facial expressions in e-learning environments. J. Univ. Shanghai Sci. Technol..

[87] Monkaresi H., Bosch N., Calvo R.A., D’Mello S.K. (2017). Automated Detection of Engagement Using Video-Based Estimation of Facial Expressions and Heart Rate. IEEE Trans. Affect. Comput..

[88] Uzelac A., Gligoric N., Krco S. (2018). System for recognizing lecture quality based on analysis of physical parameters. Telemat. Inform..

[89] Zhang S.Y., Li C. (2022). Research on Feature Fusion Speech Emotion Recognition Technology for Smart Teaching. Mob. Inf. Syst..

[90] Waheed H., Hassan S.U., Aljohani N.R., Hardman J., Alelyani S., Nawaz R. (2020). Predicting academic performance of students from VLE big data using deep learning models. Comput. Hum. Behav..

[91] Yagci M. (2022). Educational data mining: Prediction of students’ academic performance using machine learning algorithms. Smart Learn. Env..

[92] Maurya L.S., Hussain M.S., Singh S. (2021). Developing Classifiers through Machine Learning Algorithms for Student Placement Prediction Based on Academic Performance. Appl. Artif. Intell..

[93] Lin L. (2021). Smart teaching evaluation model using weighted naive bayes algorithm. J. Intell. Fuzzy Syst..

[94] Luckin R., Holmes W. (2016). Intelligence Unleashed: An Argument for AI in Education.

[95] Sun Q.N. (2021). Evaluation model of classroom teaching quality based on improved RVM algorithm and knowledge recommendation. J. Intell. Fuzzy Syst..

[96] Huang W.M. (2021). Simulation of English teaching quality evaluation model based on gaussian process machine learning. J. Intell. Fuzzy Syst..

[97] del Gobbo E., Guarino A., Cafarelli B., Grilli L., Limone P. (2023). Automatic evaluation of open-ended questions for online learning. A systematic mapping. Stud. Educ. Eval..

[98] Srikant S., Aggarwal V. A System to Grade Computer Programming Skills using Machine Learning. Proceedings of the 20th ACM SIGKDD International Conference on Knowledge Discovery and Data Mining (KDD).

[99] Zhang L.S., Huang Y.W., Yang X., Yu S.Q., Zhuang F.Z. (2022). An automatic short-answer grading model for semi-open-ended questions. Interact. Learn. Environ..

[100] Jamil F., Hameed I.A. (2023). Toward intelligent open-ended questions evaluation based on predictive optimization. Expert Syst. Appl..

[101] Botelho A., Baral S., Erickson J.A., Benachamardi P., Heffernan N.T. (2023). Leveraging natural language processing to support automated assessment and feedback for student open responses in mathematics. J. Comput. Assist. Learn..

[102] U.S. Department of Education (2017). Reimagining the Role of Technology in Education: 2017 National Education Technology Plan Update. https://www.boarddocs.com/mabe/mcpsmd/Board.nsf/files/BGYMW35CE7D7/$file/5%20US%20Dept%20of%20Ed%20Education%20Technology%20Plan%20Update%202017%20-%20excerpt.pdf.

[103] Ezzaim A., Dahbi A., Haidine A., Aqqal A. (2023). AI-Based Adaptive Learning: A Systematic Mapping of the Literature. J. Univers. Comput. Sci..

[104] Rastegarmoghadam M., Ziarati K. (2017). Improved modeling of intelligent tutoring systems using ant colony optimization. Educ. Inf. Technol..

[105] Shemshack A., Spector J.M. (2020). A systematic literature review of personalized learning terms. Smart Learn. Env..

[106] Abyaa A., Idrissi M.K., Bennani S. Predicting the learner’s personality from educational data using supervised learning. Proceedings of the 12th International Conference on Intelligent Systems—Theories and Applications (SITA), Mohammed V Univ.

[107] Pliakos K., Joo S.H., Park J.Y., Cornillie F., Vens C., Van den Noortgate W. (2019). Integrating machine learning into item response theory for addressing the cold start problem in adaptive learning systems. Comput. Educ..

[108] Tahir S., Hafeez Y., Abbas M.A., Nawaz A., Hamid B. (2022). Smart Learning Objects Retrieval for E-Learning with Contextual Recommendation based on Collaborative Filtering. Educ. Inf. Technol..

[109] Davies J.N., Verovko M., Verovko O., Solomakha I. Personalization of e-learning process using AI-powered chatbot integration. Proceedings of the International Scientific-Practical Conference.

[110] Lu Y., Chen C., Chen P., Chen X., Zhuang Z. Smart Learning Partner: An Interactive Robot for Education. Proceedings of the 19th International Conference on Artificial Intelligence in Education (AIED).

[111] Jiao F.M., Song J., Zhao X., Zhao P., Wang R. (2021). A Spoken English Teaching System Based on Speech Recognition and Machine Learning. Int. J. Emerg. Technol. Learn..

[112] Liu Y.-F., Hwang W.-Y., Su C.-H. (2022). Investigating the impact of context-awareness smart learning mechanism on EFL conversation learning. Interact. Learn. Environ..

